# Copper in cancer: from limiting nutrient to therapeutic target

**DOI:** 10.3389/fonc.2023.1209156

**Published:** 2023-06-23

**Authors:** Xiaolong Tang, Zaihua Yan, Yandong Miao, Wuhua Ha, Zheng Li, Lixia Yang, Denghai Mi

**Affiliations:** ^1^ The First Clinical Medical College, Lanzhou University, Lanzhou, Gansu, China; ^2^ The Second Department of Gastrointestinal Surgery, Affiliated Hospital of North Sichuan Medical College, Nanchong, Sichuan, China; ^3^ Department of Oncology, Yantai Affiliated Hospital of Binzhou Medical University, The Second Clinical Medical College of Binzhou Medical University, Yantai, Shandong, China; ^4^ Division of Thoracic Tumor Multimodality Treatment and Department of Radiation Oncology, Cancer Center, West China Hospital of Sichuan University, Chengdu, Sichuan, China; ^5^ Gansu Academy of Traditional Chinese Medicine, Lanzhou, Gansu, China

**Keywords:** copper metabolism, anticancer, Cu chelators, Cu ionophores, potential drugs

## Abstract

As an essential nutrient, copper’s redox properties are both beneficial and toxic to cells. Therefore, leveraging the characteristics of copper-dependent diseases or using copper toxicity to treat copper-sensitive diseases may offer new strategies for specific disease treatments. In particular, copper concentration is typically higher in cancer cells, making copper a critical limiting nutrient for cancer cell growth and proliferation. Hence, intervening in copper metabolism specific to cancer cells may become a potential tumor treatment strategy, directly impacting tumor growth and metastasis. In this review, we discuss the metabolism of copper in the body and summarize research progress on the role of copper in promoting tumor cell growth or inducing programmed cell death in tumor cells. Additionally, we elucidate the role of copper-related drugs in cancer treatment, intending to provide new perspectives for cancer treatment.

## 1 Introduction

Copper is an essential micronutrient required for regular physiological functions in the body. Within healthy individuals, the concentration of copper ranges from 50 to 120 mg, a modest amount compared to other primary trace elements such as iron and zinc. When juxtaposed with the quantities of iron and zinc, approximately 3500-4000 mg and 2000-3000 mg respectively, copper’s proportion equates to a mere 1.25% to 3.43% of iron and 1.67% to 6% of zinc (1). As a catalytic co-factor or structural component of proteins, copper plays a crucial role in many essential biological functions, including enzyme activity, oxygen transport, and cell signaling. In both the +1 and +2 oxidation states, copper ions in the body exhibit readily electron transfer and high redox activity, which are vital for enzyme-catalyzed reactions, including biological redox reactions (2). While the redox activity of copper is critical for enzyme-catalyzed reactions, this property also makes it potentially toxic. Copper’s redox activity can catalyze the production of free radicals, which may damage lipids, proteins, DNA, and other biomolecules, leading to cellular damage (3, 4). Therefore, copper has both beneficial and toxic effects on cells. Maintaining the balance of copper intake in the body is necessary to avoid potential toxicity and ensure the proper functioning of physiological processes.

In terms of the relationship between copper and tumors, on the one hand, the active metabolism of cancer cells leads to a much higher demand for nutrients than normal cells. Copper is an essential component of many biological functions and plays a critical role in the development and progression of tumors. Accumulation of copper has been shown to be associated with tumor cell proliferation (5), angiogenesis (6), and metastasis (7). Copper levels are higher in many tumor tissues than in normal tissues, and serum copper concentrations are also higher in many tumor patients than in normal individuals. Treatment with oral copper chelators has been shown to inhibit tumor growth and metastasis in animal cancer models and human patients (8). Therefore, taking advantage of the specific need of cancer cells for copper and reducing the concentration of copper ions in tumor tissues through drug intervention could be a breakthrough in tumor treatment. On the other hand, numerous studies have shown that high concentrations of copper have potential toxicity to tumor cells (9–11). Under high copper concentrations, free intracellular copper ions accumulate and inhibit enzyme function by oxidizing cysteines in iron-sulphur cluster proteins or react chemically with hydrogen peroxide to generate highly reactive hydroxyl radicals, thereby inducing cellular damage (12). Recently, Tsvetkov et al. have pointed out that monovalent copper ions (Cu^+^) can directly bind to the lipoylated components of the tricarboxylic acid (TCA) cycle, causing aggregation of lipoylated proteins, leading to protein toxicity stress and ultimately inducing cell death in tumor cells (13). To prevent damage caused by excessive copper concentration, organisms have evolved complex copper transport and copper-binding proteins to control copper uptake, intracellular transport, storage, and efflux and prevent the formation of highly toxic Cu^+^ within cells. After completion of growth and development, the organism can maintain systemic copper homeostasis by controlling the balance between copper absorption and excretion (14). Therefore, based on the high copper concentration in tumor tissues, depleting copper from tumor tissues or inducing excessive copper accumulation in tumor cells by disrupting their copper homeostasis using drugs and other means may induce tumor cell death in various ways. This approach may provide a novel strategy for tumor therapy.

Hence, the characteristic of copper metabolism in cancer cells may be their specific weakness compared to normal tissues (15). Copper accumulation in tumor cells has two biological properties. On the one hand, it can promote tumor development by promoting cell proliferation, metastasis, and angiogenesis. On the other hand, it can induce programmed cell death in tumor cells, thereby inhibiting tumor development. Given this, it is necessary to collate relevant studies and results to present a clearer picture of the overall role of copper in tumors and explore its potential as a tumor indicator and target.

## 2 Physiological effects and metabolic processes of copper

Copper plays an essential role in cellular metabolism, participating in many physiological processes such as the mitochondrial respiratory chain, antioxidant reactions, and biosynthesis of biomolecules. The metabolic process of copper is essential for maintaining the normal physiological function, which involves the participation and regulation of various copper transport proteins. Both too low or too high copper levels can harm cells, so they must be maintained within an appropriate range. Studies have shown that the occurrence and development of various diseases are related to a copper imbalance in the body, including Menkes disease (16), Wilson’s disease (17), neurodegenerative diseases, and cancer (18). Copper homeostasis in living organisms mainly depends on the regulation of copper uptake, transport, storage, and excretion by the body and cells, as well as the dynamic balance of the amount and distribution of copper ions in different forms.

### 2.1 Physiological functions of copper

As a vital trace element, copper plays a crucial role in many physiological activities in the body. In biological systems, copper ions mainly exist in two forms: the reduced form of cuprous ion (Cu^+^) and the oxidized form of cupric ion (Cu^2+^). The extracellular environment mainly contains Cu^2+^, while the intracellular environment mainly contains Cu^+^ (19, 20). Cu^2+^ functions by binding and regulating growth factors and cell membrane receptors in the extracellular environment, while Cu^+^ exists on the cell membrane and directly regulates kinase activity to alter the activation state of growth factor membrane receptors through structural modification and inhibition of phosphatase function. Cu^+^ can also bind to transcription factors in the cell nucleus, thereby affecting gene expression and subsequent protein synthesis (21). Copper is an essential catalytic cofactor in redox protein chemistry. It plays a crucial role in carrying out the biological functions required for growth and development, making it extremely important in eukaryotes (19, 22).

Currently, about 54 copper-binding proteins have been discovered, with approximately half being enzymes, 12 being copper transporters, and one being a transcription factor antioxidant 1 copper chaperone (ATOX1) (23). The enzyme types in these copper-binding proteins mainly include copper-zinc superoxide dismutase, cytochrome oxidase, dopamine-beta-hydroxylase, tyrosinase, and lysyl oxidase, which participate in many essential biological processes such as neurotransmitter synthesis, mitochondrial respiration, extracellular matrix crosslinking, pigmentation, and antioxidant defense (23, 24). Copper is mainly stored and distributed within cells by mitochondria. The cytochrome c oxidase copper chaperone COX17 (COX17), located on the mitochondrial membrane, can bind with 1-4 Cu^+^ ions. It is responsible for transporting Cu^+^ from the cytoplasm to the intermembrane space of mitochondria to the synthesis of cytochrome c oxidase 1 (SCO1) and cytochrome c oxidase (CCO), thus maintaining the normal function of enzymes in the respiratory chain (19).

In mitochondria, the electron transport chain (ETC) is the primary process required to maintain normal cell metabolism, including multiple components such as NADH dehydrogenase (complex I), succinate dehydrogenase (complex II), ubiquinol-cytochrome c reductase (complex III), cytochrome c oxidase (complex IV), and electron carriers. The correct assembly and functional operation of these components is all dependent on the involvement of copper (19, 25–28). In addition, copper/zinc superoxide dismutase 1 (SOD1) also relies on the presence of copper. SOD1 is located in the mitochondrial inner membrane and cytoplasmic solute and can mitigate the reactive oxygen species (ROS) produced by ETC (29, 30). Iron not only plays an important role in iron-sulfur cluster protein assembly and heme biosynthesis but also is a critical element in maintaining cellular metabolic balance and function within mitochondria (31). At the same time, copper acts as an auxiliary factor for ferroxidases to regulate the ETC on the mitochondrial membrane by affecting mitochondrial iron uptake (32, 33). In addition to indirectly affecting iron uptake, copper may also directly regulate the assembly or degradation of respiratory complex IV within the ETC. Complex IV can act as a metal sensor to regulate respiratory frequency, and the lack of copper can reduce its expression and activity (34–37). These findings suggest that managing cellular copper levels through metabolic reprogramming is a simple way to switch the main energy metabolism pathway of cells between glycolysis and oxidative phosphorylation (OXPHOS) to control cell fate. At the mitochondrial level, the use of copper chelators can lead to a decrease in cellular COX levels, mitochondrial respiratory function, and ROS levels while activating glycolysis (36, 38). In addition, studies have found that excess copper in cells can activate cyclin-dependent kinases (CDK2) and cell cycle proteins, promoting cell division (39). ATOX1, when bound to Cu^+^, can also act as a transcription factor, leading to the expression of G1/S-specific cyclin D1, inducing cell proliferation (40).

Therefore, copper is an essential trace element for metabolism in the body. It plays a crucial role in maintaining normal growth and development of the body, as well as the regular operation of various physiological processes. Especially in terms of cellular respiratory function, copper plays an extremely important role.

### 2.2 Copper absorption, distribution, and excretion

Copper is the third most abundant essential trace element in the human body, following zinc and iron. The average copper content in an adult human body is about 80mg, with the highest concentrations found in tissues such as the eyes, heart, liver, and brain (41). Typically, the serum copper concentration in healthy adults ranges from 70-110 μg/dL (42). About 5% of the total copper content in the body is found in the serum, with approximately 95% bound to ceruloplasmin (CP). Copper is mainly absorbed into the body through the small intestine (43). According to recommendations, adults should ingest 0.9mg of copper daily. The average diet of most individuals can meet or exceed this requirement (44). ATPase copper transporting alpha (ATP7A) in the intestinal epithelial cells is the critical protein for transporting copper ions from the intestine into the body. ATP7A transports copper to the portal vein and then through serum proteins such as albumin for transportation to the liver. When copper ions exceed standard requirements, they are bound to metallothionein 1 (MT1) and metallothionein 2 (MT2) and stored in liver cells (45). ATPase copper transporting beta (ATP7B) primarily functions in the efflux of copper ions from cells (46). Copper stored in liver cells can be released into the bloodstream for further distribution or transported to bile for excretion. When the copper ion content is high in liver cells, the ATOX1 binds with excess copper ions and transports them to the N-terminal metal-binding region of ATP7B, which is then excreted through the bile duct membrane. The body maintains systemic copper homeostasis by coordinating gastrointestinal absorption and bile excretion balance (14). In the circulatory system, copper ions bind with CP and albumin for transport throughout the body (47, 48). CP is the leading carrier for copper transport in the circulatory system. As CP rapidly degrades without metal, its abundance in plasma serves as a biological marker for systemic copper deficiency (49). The liver is one of the most crucial copper metabolic organs in the body, playing a central role in regulating systemic copper homeostasis. The liver metabolizes and excretes copper, participating in many biological processes, including cellular respiration, antioxidant defense, iron homeostasis, and neuropeptide processing (50).

Before extracellular Cu^2+^ can be absorbed by cells, they need to be reduced to the monovalent reduced form of Cu^+^ by metal reductases on the cell surface and then taken up by the cells through the solute carrier family 31 member 1 (SLC31A1, also known as CTR1) (43). Once inside the cell, copper is captured by multiple molecular chaperones and transported to the relevant copper-dependent proteins. These chaperone proteins include the SCO1, synthesis of cytochrome c oxidase 2 (SCO2), cytochrome c oxidase copper chaperone COX11 (COX11), copper chaperone for superoxide dismutase (CCS), COX17, and ATOX1. SCO1, SCO2, and COX11 are used to direct copper transport to various metalloenzymes and copper-dependent ATPases with copper output and metal chaperone functions, including ATP7A and ATP7B. The CCS transports copper ions to superoxide dismutase 1 (SOD1) (19). The COX17 transports copper to CCO (51, 52). The ATOX1 also transports copper to ATP7A and ATP7B across the Golgi network and regulates copper subcellular distribution through the complex transport mechanisms of the Golgi network. This mechanism is responsible for providing copper ions to secreted copper enzymes, such as lysyl oxidase (LOX) and CP, in order to eliminate excess copper ions within the cell.

The copper within cells must be maintained at very low levels, with the vast majority bound to proteins (53). Unbound Cu^1+^ in the cell are strong oxidants, catalyzing the generation of highly reactive carbonyl free radicals that can damage proteins, DNA, and lipids (54). Additionally, excess copper ions may displace other metals from homologous ligands in metalloproteins, leading to improper protein conformation or impaired enzymatic activity (55). Because the presence of free copper can potentially be toxic to cells, the concentration of free copper in the cytoplasm must be kept at very low levels in the body, estimated to be between 10^-15^ M to 10^-21^ M or less than one free copper ion per cell (15). In order to prevent potential harm caused by the accumulation of copper in the body, various copper-binding proteins are employed, along with the regulation of copper absorption and excretion, to maintain low levels of copper. MT1 and MT2, which are rich in cysteine, can prevent copper toxicity by irreversible chelation within metal-sulfur salt clusters and can be induced by excess copper or other metals transcriptionally (56). Additionally, the antioxidant glutathione (GSH) can also bind copper ions in the cell solute to limit fluctuations in copper ion concentration (57). Compared to metallothioneins, the copper bound to GSH can easily exchange with higher affinity ligands such as metallochaperones. The cytoplasmic concentration of GSH is estimated to be in the millimolar range, which is much higher than the steady-state copper concentration (57). This phenomenon allows GSH to act as a buffer for copper in the cellular solute. It not only prevents the formation of free copper ions but also maintains a negative concentration gradient across the membrane, driving the uptake of copper by the copper transporter protein, such as CTR1 (58). With the combined action of these proteins, intracellular copper ions are maintained at an appropriate level. Copper-dependent enzymes are also able to maintain their normal metalation and function properly. These copper-dependent enzymes include cytochrome c oxidase, superoxide dismutase, and various oxygenases and oxidases such as tyrosinase, lysyl oxidase, dopamine beta-hydroxylase, and copper amine oxidase (53).

**Figure 1:**
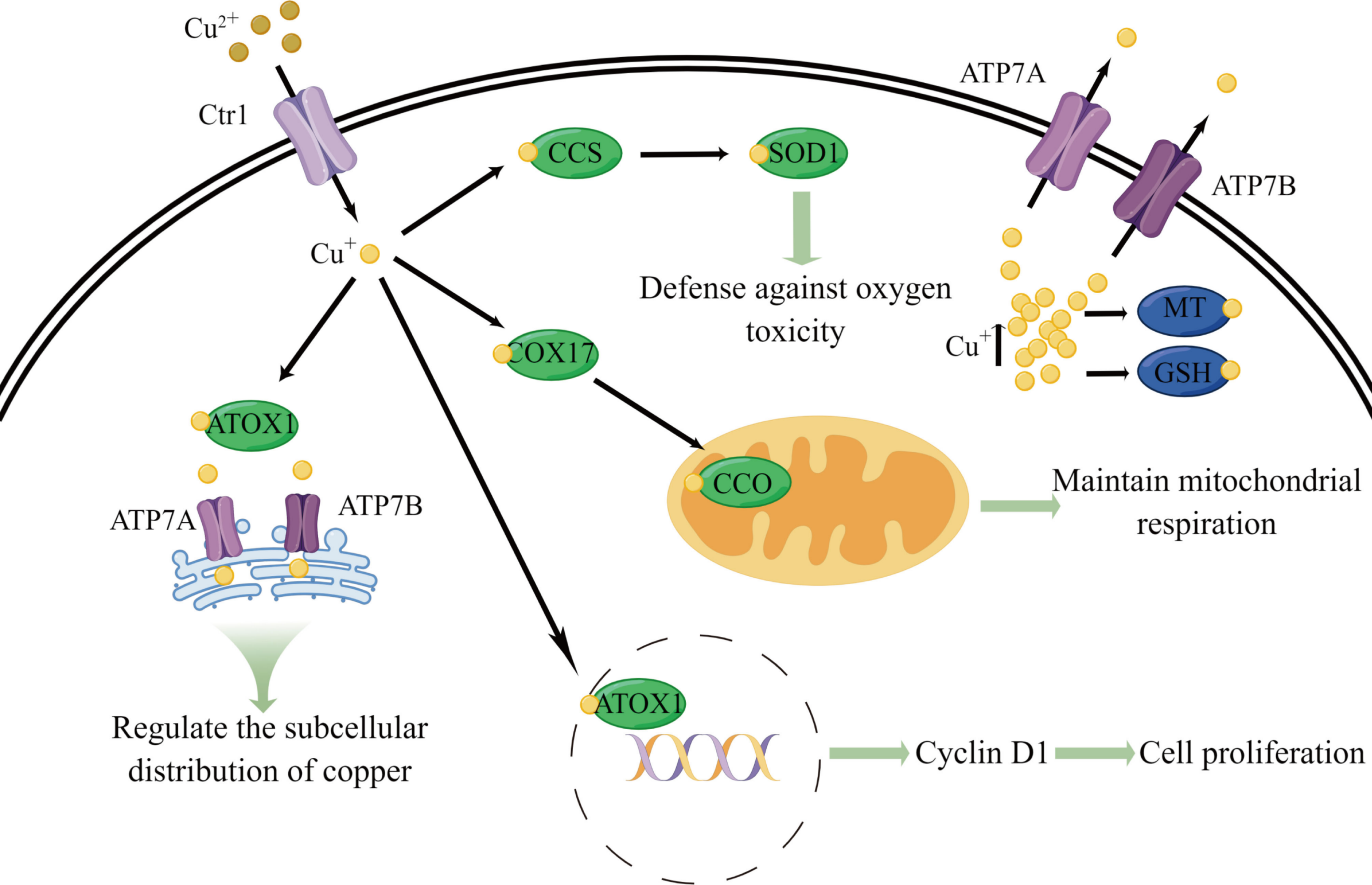
Schematic diagram of some essential physiological functions and metabolic processes of copper. Copper is primarily transported into cells by CTR1. Upon entering the cell, copper is transferred to CCS, COX17, and ATOX1. CCS delivers copper to SOD1, which is involved in defense against oxygen toxicity. COX17 transfers copper to CCO, which participates in maintaining mitochondrial respiration. ATOX1 can carries copper into the cell nucleus, leading to the expression of G1/S-specific cyclin D1 and inducing cell proliferation. ATOX1 also transports copper to ATP7A and ATP7B across the Golgi network and regulates copper subcellular distribution through the complex transport mechanisms of the Golgi network. Excess copper in the cell is bound by MT and GSH to prevent copper toxicity. In addition, ATP7A and ATP7B primarily function in the efflux of copper ions from cells.

In summary, to maintain copper balance in the body, a complex system of copper metabolism regulation is employed, including absorption, transport, and excretion of copper, as well as its uptake and utilization within cells at multiple levels. **Figure 1** illustrates some of the essential physiological functions and metabolic processes of copper. The coordinated actions of these mechanisms ensure proper distribution of copper ions in the body while preventing harmful effects such as hydroxyl free radical formation from free copper ions, thus protecting cells from damage. Only in this way can normal cell function be maintained, and the toxic effects of excess or deficient copper avoided (59).

## 3 Mechanisms of copper in promoting tumorigenesis

Elevated copper concentrations have been observed in a multitude of cancer types (8). The maintenance of copper equilibrium in tumor cells involves a complex interplay of several factors. The causes for the amplified copper content within tumors may be twofold. Primarily, tumors, particularly rapidly growing ones, have high metabolic demands. Copper is a cofactor for multiple enzymes involved in cellular energy metabolism, such as CCO, and in antioxidant defenses, such as superoxide dismutase (8, 60). Thus, the demand for copper in these processes may be increased in cancer cells. Secondarily, in tissues suffering from hypoxia, an upregulation of CTR1 has been noted (61). This condition of oxygen deficiency is often associated with tumors. Hypoxia-inducible factor 1-alpha (HIF-1α) may indirectly stabilize and activate the transcription of numerous genes involved in copper metabolism, including those controlling CTR1, thereby contributing to higher copper levels in tumor cells (62). Indeed, CTR1 has been definitively found to be upregulated in a wide variety of tumors (8).

### 3.1 Copper and tumor growth

Copper ion redox function is essential for biochemical reactions in living organisms. Therefore, imbalances in copper homeostasis are associated with a variety of diseases, including diabetes, neurological disorders, and cancer (63). Studies have indicated that tumors often exhibit copper imbalances, and alterations in copper ion concentrations can affect mitochondrial respiration, glycolysis, insulin resistance, and lipid metabolism (64–66). Wilson’s disease patients and animal models have shown an increased incidence of liver cancer, suggesting that aberrant copper accumulation may promote the malignant transformation of liver cells via unknown mechanisms (67). The relationship between copper metabolism and tumor development has been extensively investigated. Cancer cells require higher levels of copper to meet their energy demands for rapid proliferation compared to normal cells (68–70). This may be because copper serves as a cofactor for cytochrome c oxidase in the mitochondria (8), which is involved in electron transport and ATP synthesis in the mitochondrial respiratory chain. Exposure to high levels of copper (the maximum allowable level in public water is 1.3 mg/L) has been shown to promote tumor progression (71). The levels of copper in blood and tissues are significantly elevated in various types of cancer patients, including liver cancer (72), colorectal cancer (73), lung cancer (74), and breast cancer (75). Following successful tumor removal or alleviation, copper levels in the blood return to normal. Gene expression analysis has revealed significant changes in different copper-binding or copper-sensitive proteins in colorectal cancer (76) and breast cancer (77), suggesting a close association between copper homeostasis dysregulation and cancer occurrence, development, and metastasis. Cancer cells typically express high levels of the transmembrane transport protein CTR1 to obtain more copper. Even in conditions of increased glycolysis, the addition of copper chelators can significantly reduce ATP production in cancer cells, indicating their continued reliance on mitochondrial respiration and OXPHOS for energy (64). Elevated copper levels may therefore play a role as a limiting nutrient in tumorigenesis and development by regulating ATP production through OXPHOS to meet cancer cells’ rapid proliferation demands (64).

Tumor protein p53 (P53) protein is a crucial tumor suppressor protein that regulates the cell cycle, inhibits the division of DNA-damaged cells, and promotes cell apoptosis. Unfortunately, P53 frequently undergoes mutations in tumor cells, rendering it unable to function properly (78). During the normal function of P53, it requires binding with a single zinc ion. The presence of copper ions may disrupt this process, resulting in the inability of the P53 protein to function correctly. This disruption of P53 protein function could be one aspect of the relationship between copper and the mechanism of tumor development (79, 80). Studies have shown that high concentrations of copper elements play a crucial role in the progression from non-alcoholic fatty liver disease (NAFLD)-induced liver cirrhosis to hepatocellular carcinoma (HCC) (81). It is noteworthy that the phenomenon of elevated serum copper levels is particularly prominent in both NAFLD-induced liver cirrhosis patients and HCC patients. A high concentration of copper element can stimulate the proliferation, migration, and invasion of liver cancer cells by modulating the v-myc avian myelocytomatosis viral oncogene homolog (MYC)/CTR1 axis. Additionally, MYC protein can bind to specific regions on the CTR1 promoter and regulate its transcription, thereby increasing the concentration of the copper element within liver cancer cells. Therefore, the expression of CTR1 and MYC protein gradually increases during the progression from NAFLD-induced liver fibrosis to HCC (81). Knocking out the copper transporter protein CTR1 can effectively reduce the phosphorylation levels of Extracellular Signal-Regulated Kinase 1/2 (ERK1/2) in melanoma cells, which is responsive to cell proliferation, thus inhibiting melanoma cell proliferation. In contrast, cells treated with copper show increased phosphorylation of the upstream receptor tyrosine kinases, such as tropomyosin receptor kinase B (TRKB), epidermal growth factor receptor (EGFR), and hepatocyte growth factor receptor (MET) in the mitogen-activated protein kinases (MAPK) signaling pathway (82–84). In addition, copper can affect mitogen-activated protein kinase kinase 1 (MEK1) and mitogen-activated protein kinase kinase 2 (MEK2) and enhance their phosphorylation of ERK1 and ERK2 in a dose-dependent manner, further promoting tumor cell proliferation (85). In a chemically induced rat model of breast cancer, model rats administered copper orally daily were more likely to experience rapid cancer cell growth than model rats without copper supplementation (86). Similarly, copper intake can accelerate the growth rate of pancreatic islet cell carcinoma in mice (64). When the transport of Cu^2+^ is blocked, cells produce oxidative stress reactions that lead to a decrease in ATP levels while activating AMP-activated protein kinase to decrease fat synthesis and inhibit tumor cell proliferation (87).

Copper is an essential co-factor for the autophagy kinases, namely unc-51 like autophagy activating kinase 1 (ULK1) and unc-51 like autophagy activating kinase 2 (ULK2). When intracellular copper levels increase, it can promote the activity of ULK1 and ULK2 kinases, thereby increasing autophagy flux and promoting the growth and survival of lung tumor cells (88). In addition, the accumulation of copper in the liver in Wilson’s disease is highly correlated with the activation of autophagy, which confirms the correlation between copper and autophagy (89). In a mouse model of colorectal cancer, tumor cells with Kirsten rat sarcoma viral oncogene homolog (KRAS) gene mutations can use macropinocytosis to acquire more copper, which seems to be essential for supporting tumor growth. In contrast, reduced copper bioavailability inhibits the proliferation of KRAS mutant tumor cells (90).

The above studies suggest that elevated copper concentrations can promote tumor growth by enhancing mitochondrial energy metabolism, causing misfolding of tumor-associated proteins, inducing upregulation of tumor-associated pathway signaling and affecting autophagy kinase activity.

### 3.2 Copper and tumor angiogenesis

The role of copper in promoting angiogenesis was first reported by McAuslan et al. in 1979. They found that copper can induce endothelial cell migration, which is an early stage in the formation of blood vessels (91). Subsequent studies showed that copper levels increase with the formation of new blood vessels in the rabbit cornea (92). Further research has demonstrated that the addition of copper salts is sufficient to induce blood vessel formation (93). In this model, if these rabbits were deficient in copper, corneal neointima formation would also be inhibited (92, 93). There are multiple molecular mechanisms by which copper induces angiogenesis. On the one hand, copper can directly bind to growth factors related to angiogenesis, enhancing their affinity to endothelial cells (94). On the other hand, copper ions can increase the production and medium of nitric oxide, leading to the activation of tumor-promoting angiogenesis signals (95). In addition, copper’s regulatory effect on angiogenic factors such as fibroblast growth factor (FGF) and interleukin-1 alpha (IL-1α) is also crucial, as the secretion of these factors requires the involvement of copper-dependent multi-protein complexes (96, 97). Copper deficiency can inhibit nuclear factor kappa-light-chain-enhancer of activated B cells (NF-κB) activity, thereby reducing the expression of 5 pro-angiogenic mediators, namely vascular endothelial growth factor (VEGF), fibroblast growth factor 2 (FGF2), IL-1α, interleukin-6 (IL-6), and interleukin-8 (IL-8) (98). In addition, the copper-dependent transcription factor ATOX1 can be involved in tumor angiogenesis and vascular remodeling by regulating the platelet-derived growth factor (PDGF) signaling pathway (99). HIF-1α plays an essential role in promoting tumor growth by stimulating angiogenesis through various pathways, including the induction of VEGF (100). Research has shown that copper can regulate the generation of transcriptional complexes by interacting with HIF-1α, thereby regulating the transcriptional activity of HIF-1α (101). Additionally, copper can selectively regulate the binding of HIF-1α to affected gene promoters, thereby affecting its transcriptional activity (102). Clinical studies have demonstrated a significant positive correlation between serum copper levels and HIF-1α levels in patients with liver cancer, suggesting that copper accumulation may lead to the activation of HIF-1α and promote the development of liver cancer (103). DCA50, a novel inhibitor of the intracellular copper chaperone proteins ATOX1 and CCS, can induce an increase in cellular copper content and subcellular redistribution of copper by interfering with cellular copper homeostasis, thereby inhibiting tumor angiogenesis and inducing apoptosis in triple-negative breast cancer cells (104).

The above studies indicate that copper plays a vital role in tumor angiogenesis. Its mechanism of action includes promoting tumor angiogenesis by affecting HIF-1α activity and regulating the secretion of various angiogenic factors. Lowering the copper concentration in tumor tissues or inhibiting copper transport within tumor cells can effectively inhibit tumor angiogenesis, making it a potential therapeutic strategy for cancer.

### 3.3 Copper and tumor metastasis

Copper not only affects the proliferation and angiogenesis of tumor cells but also directly participates in the invasion and metastasis of cancer cells. Copper-associated LOX catalyzes the crosslinking of collagen and elastin, promoting the maturation of the extracellular matrix (105). In tumors, the expression and activity of LOX undergo changes, and cancer cells reshape the extracellular matrix by secreting LOX, creating an environment more conducive to cancer cell metastasis (106). The activity of LOX and lysyl oxidase-like (LOXL) proteins depends on copper (105, 107). The copper transport protein ATP7A plays an essential role in the regular enzymatic activity of LOX and LOXL. In a mouse model of breast cancer *in situ*, silencing of ATP7A inhibited the activity of LOX, thus rendering the LOX-dependent metastatic mechanism ineffective in breast cancer cell lines (108). The high expression of the lysyl oxidase-like 2 (LOXL2) gene is closely associated with the high invasiveness of tumors and the metastasis and prognosis of female breast cancer (109, 110). Studies have also found that LOXL2 can downregulate E-cadherin and tight junction proteins, leading to epithelial-mesenchymal transition (111). These findings suggest that targeting copper to inhibit the activity of LOX and LOXL can effectively suppress cancer cell invasion and metastasis. The mediator of cell motility 1 (MEMO1) is a copper-dependent oxidoreductase that plays a critical role in cell motility. Studies have shown that MEMO1 promotes breast cancer cell invasion and metastasis. Treatment with the copper chelator tetrathiomolybdate (TTM) reduces Cu^2+^ levels, decreases MEMO1 expression, delays angiogenesis, and inhibits breast cancer cell invasion and metastasis (112). In MCF-7 cells, copper depletion by chemical chelation of copper ions or knockdown of copper transporter CTR1 inhibits tumor cell metastasis and reduces the expression of epithelial-mesenchymal transition (EMT) mesenchymal gene markers (113). In addition, copper is also involved in the binding of HIF-1α to the hypoxia response element (HRE) sequence of target genes, which requires the copper molecular chaperone CCS, and activates EMT-related genes such as snail family transcriptional repressor 1 (SNAIL1), twist-related protein 1 (TWIST1), snail family transcriptional repressor 2 (SNAIL2), zinc finger E-box-binding homeobox 1 (ZEB1), zinc finger E-box-binding homeobox 2 (ZEB2), and E12/E47 (101).

Therefore, copper can affect the activity of LOX and LOXL enzymes, the expression of MEMO1, and the binding of HIF-1α to target genes with HRE sequences (hypoxia response elements), thereby regulating the expression of EMT-related genes and promoting tumor cell metastasis. Thus, targeting copper in therapy can block this pathway and inhibit tumor cell metastasis.

### 3.4 Copper and metabolic reprogramming of tumor

The metabolic demands of tumor cells are reprogrammed to support proliferation and minimize apoptosis by adapting to different oxygen levels. Glycolysis is the process of breaking down sugar molecules into smaller ones to generate energy. In aerobic conditions, the glycolysis of tumor cells increases, glucose uptake is enhanced, lactate production is increased, and oxidative phosphorylation is weakened. This phenomenon is known as the “Warburg effect” or “aerobic glycolysis” (114). This metabolic reprogramming is a crucial feature of tumor development and has been widely studied in clinical settings. It provides tumor cells sufficient energy and nutrients to adapt to various environmental stresses, including hypoxia and starvation, which are tightly linked to cell proliferation and survival. Aerobic glycolysis gives cancer cells a competitive advantage over normal cells, making them more likely to survive and reproduce. This adaptive response may be caused by mutations that deactivate or activate tumor suppressor genes or oncogenes under hypoxic conditions. Hypoxia is an effective inducer of overexpression of glycolysis-related genes in tumor cells to maintain their energy sources. Therefore, a higher rate of glycolysis in cancer cells can be achieved by increasing transcription, followed by the translation of glycolysis genes and glucose transporter, and inhibiting oxidative phosphorylation. HIF-1α is a major regulatory factor in the hypoxia response, playing a critical role in the regulation of the glycolysis process. There is evidence that HIF-1α plays a crucial role in the carcinogenesis process. Additionally, as previously mentioned, there is a close relationship between excess copper and overexpression of HIF-1α (101, 102). Under conditions of sufficient oxygen, the von Hippel-Lindau protein (VHL) promotes the degradation of HIF-1α by mediating ubiquitination. However, in hypoxic environments, VHL cannot mediate the ubiquitination of HIF-1α due to the lack of prolyl hydroxylase domain (PHD) proteins to multi-ubiquitinate or hydroxylate proline residues, resulting in metabolic changes (115). Some studies suggest that copper can inhibit PHD, thus enhancing the function and activity of HIF-1α by interfering with PHD activation (116). The copper-mediated inhibition of PHD and the resulting enhancement of HIF-1α function and activity will enhance downstream transcription of HIF-1α targeted genes, leading to metabolic reprogramming that enables tumors to better adapt to hypoxic environments. However, there are conflicting reports, as mentioned earlier, that copper is also an essential component of respiratory complex IV, and the amount of copper directly affects the activity of complex IV, thus affecting the overall function of the ETC (34–37). At the mitochondrial level, using copper chelators can lead to a decrease in COX levels and mitochondrial respiration levels and activate glycolysis (36, 38).

Therefore, the role of copper in tumor metabolic reprogramming may not be a simple linear relationship but a complex interplay of multiple mechanisms. Further research is needed to investigate the mechanisms underlying the role of copper in tumor metabolic reprogramming in order to develop more effective treatment strategies.

### 3.5 Copper and the tumor immune microenvironment

The occurrence and development of tumors are closely related to factors such as immune cell infiltration, immune modulation, cytokines, immune suppressive cells, and immune evasion in the tumor immune microenvironment (TIME) (117). Copper is closely related to the tumor immune microenvironment and various immune cells. Studies have shown that copper ion levels in mesothelioma affect the infiltration of CD4+ T cells, with an increase in CD4+ T cells when copper ion concentration significantly decreases, but the infiltration level of CD8+ T cells is not affected (118). This phenomenon indicates that copper ions may have different regulatory effects on different types of immune cells, further illustrating the close relationship between copper and the tumor immune microenvironment. Recent studies have shown that the copper ion concentration in tumor cells is closely related to the expression of programmed death-ligand 1 (PD-L1). In addition, the expression of copper transporter CTR1 and PD-L1 in tumor tissue also shows a high correlation. Studies have found that copper supplementation can promote the expression of the PD-L1 gene and protein levels in tumor cells (119). These findings indicate that the regulation of copper ion concentration may be a new mechanism for tumor immune evasion, as PD-L1 is an important molecule that inhibits T-cell immune responses. Therefore, this study provides new insights into the exploration of the mechanism of tumor immune evasion. Another study has shown that myeloid-derived suppressor cells (MDSC) from bone marrow can suppress immune responses and promote tumor development. Copper chelators can mediate MDSC cell apoptosis *in vivo*, leading to the differentiation of tumor-associated CD4+ T cells into helper T cell type 1 and participation in immune responses (120). In addition, copper ions may be involved in maintaining the pro-inflammatory phenotype of macrophages, and copper clearance in the tumor microenvironment can lead to macrophage polarization towards the M2 phenotype (121). The above studies suggest that copper ions play a definite role in the TIME and may be important in tumor immunotherapy. However, there are few studies on the regulation of copper in the TIME, and the relevant molecular mechanisms are unclear.

In summary, copper ion concentration may play a crucial role in the TIME, and these findings suggest that targeting copper ions could be a promising adjunctive immunotherapy approach. With a further understanding of the relationship between copper and the tumor immune microenvironment, we can explore more effective strategies for targeting copper ions and apply them in clinical practice to improve the success rate and efficacy of tumor immunotherapy.

**Figure 2:**
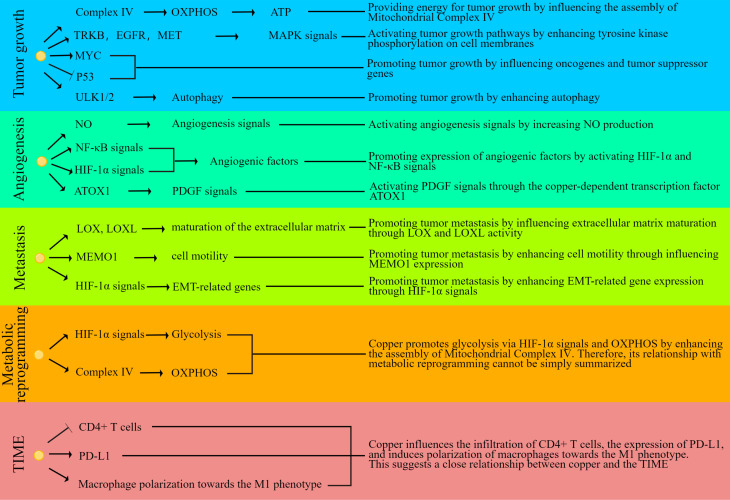
Schematic diagram of molecular mechanisms linking copper with oncology. The small yellow dots represent copper, arrows indicate promotion or stimulation, and blunt end line signify inhibition or suppression.

Taken together, copper may potentially contribute to tumor progression at multiple levels, including tumor growth, angiogenesis, metastasis, metabolic reprogramming, and TIME. **Figure 2** illustrates some of the fundamental mechanisms that underlie the relationship between copper and tumors. Consequently, these insights provide a nuanced understanding of the multifaceted role of copper in oncology, and they point towards potential therapeutic interventions that could harness the interplay between copper homeostasis and cancer progression for improved patient outcomes.

## 4 Mechanism of cell death triggered by copper overload

Copper has a U-shaped dose-response relationship and exhibits bidirectional regulation (53, 122). When copper accumulates excessively or is improperly transported, the disruption of copper homeostasis causes cellular toxicity when copper concentrations in cells exceed the threshold that homeostatic mechanisms can tolerate (123). Copper metabolism disorders are closely related to mechanisms such as cell death and neural damage. For example, Menkes disease (16), which is caused by copper deficiency due to a mutation in the ATP7A gene, and Wilson’s disease (46, 124), which is caused by liver copper accumulation due to a mutation in the ATP7B gene, are typical examples of copper imbalance-induced diseases in the body. Severe copper deficiency may lead to various types of damage, such as energy generation impairment caused by dysfunction of CCO in mitochondria. In the nervous system, copper is critical for the function of various neurons, as it is involved in myelin formation and is closely related to the regulation of synaptic activity and signal cascades induced by neurotrophic factors (125). In addition to Menkes disease, which is caused by severe copper deficiency, there is ample evidence that other neurodegenerative diseases, such as Parkinson’s disease, Alzheimer’s disease, amyotrophic lateral sclerosis, and Huntington’s disease, are also associated with disruption of copper pathways (126). Excessive intracellular copper concentrations can cause various forms of cellular damage. Excessive copper intake from the environment may also disrupt the copper metabolic balance within cells, leading to cellular toxicity and damage to the body (19). This cellular toxicity may result from improper binding of copper to protein sites, leading to protein misfolding, aggregation, and loss of function. Research has shown that when normal human bronchial epithelial cells are exposed to copper, it activates the expression of heat shock response genes, ubiquitin-related genes, and autophagy regulatory genes and causes degradation of proteasomes, resulting in an increase of misfolded proteins (48). The regulation of programmed cell death (PCD) is a crucial determinant of cell fate (127), and studies have demonstrated that copper overload can induce various forms of PCD in cells. There are conflicting views on the mechanisms by which copper induces PCD, with some scholars suggesting that copper overload-induced cell death is mainly through oxidative stress-induced apoptosis (128). Nagai, Masazumi et al. also showed that elesclomol transports divalent copper to mitochondria, where it is reduced to the more toxic monovalent copper, promoting a mitochondrial ROS response that leads to apoptosis (129). However, an important paper by Tsvetkov et al. has pointed out that copper-induced cell death does not depend on caspases. That is, it does not depend on the apoptotic pathway (13). They found that using the potent copper ionophore elesclomol as the test substance, elesclomol-induced human renal smooth muscle tumor G402 cell death did not involve the activation of caspase-3. In addition, other cell death inhibitors were also unable to eliminate elesclomol-induced cell death (13), indicating that the mechanism of elesclomol-induced cell death is different from known mechanisms of cell death. Therefore, in order to clarify the mechanisms underlying copper-mediated cell death, further research is needed to consolidate and analyze relevant studies.

### 4.1 Copper overload triggers apoptosis

Apoptosis, a type of programmed cell death, is marked by substantial alterations in cell morphology and the activation of specific caspase and mitochondrial control pathways (130). Preliminary research indicated that an excess of copper might trigger apoptosis. Recent research has found that disulfiram (DSF) can significantly increase the concentration of copper ions in melanoma cells, inducing complex redox reactions and leading to cell apoptosis (131). Elesclomol has also been found to cause apoptosis in melanoma cells via oxidative stress, though the role of copper in this process remains unclear (132). Some studies have shown that excessive copper can trigger stress-induced damage to organelles such as mitochondria and endoplasmic reticulum, which are associated with intrinsic apoptosis. Reversing such damage to these organelles can mitigate copper-induced apoptosis (48, 53). Copper can induce cell apoptosis through multiple molecular mechanisms. Treatment of mouse liver cells with copper sulfate resulted in significant upregulation of protein expression in the CHOP, JNK, and caspase-12 pathways, leading to the activation of apoptosis-related pathways and ultimately resulting in vacuolization of liver cells (133). Later experiments have found that adding high doses of copper can increase reactive oxygen species and protein carbonylation levels, decrease glutathione levels and SOD enzyme activity, leading to depolarization of mitochondrial membrane potential, the release of cytochrome c, elevated levels of active caspase-9 and caspase-3, increased levels of Bcl2-antagonist/killer 1 (Bak1) and Bcl2-associated X protein (Bax), and decreased levels of B-cell lymphoma 2 (Bcl2), ultimately resulting in cell apoptosis (134). Copper can also induce cell apoptosis through the autophagy pathway. Treatment of mouse mononuclear macrophages with copper sulfate increases mitochondrial reactive oxygen species, activates the Akt/mTOR signaling pathway to promote autophagosome formation, and upregulates the expression of cleaved caspase3/8/9 polymerase, leading to cell apoptosis (135). Furthermore, excessive copper in chicken liver cells leads to varying degrees of nuclear membrane damage, mitochondrial swelling and vacuolization, upregulation of caspase-3, cytochrome c, Bax, and Bak1 expression, downregulation of Bcl2, and increased expression of P53, indicating that copper may induce cell apoptosis by damaging mitochondria (136). In addition, copper can induce endoplasmic reticulum stress-mediated cell apoptosis. Treatment of zebrafish embryos with copper nanoparticles and copper sulfate results in the reduced inner mitochondrial membrane and increased vesicle formation, disordered endoplasmic reticulum structure, increased reactive oxygen species, elevated levels of active caspase-3, and decreased levels of Bcl2, leading to cell apoptosis and retinal developmental defects. Copper overload-induced endoplasmic reticulum stress is associated with exposure to copper nanoparticles and copper ion release. However, in mutants of copper transport proteins Cox17-/- and Atp7a-/-, there is a significant reduction in reactive oxygen species and unfolded protein, leading to the relief of apoptosis (137). It is worth mentioning that some studies have indicated that copper-induced cell death does not depend on caspase activation (138). In glioblastoma, elesclomol has been shown to induce non-apoptotic, copper-dependent cell death by promoting mitochondrial ROS production (139). Thus, copper may not solely induce cell death in the form of apoptosis.

Based on the studies mentioned above, it is evident that copper overload can induce cell apoptosis through multiple molecular pathways, including the mitochondrial control pathway, endoplasmic reticulum stress-mediated pathway, and autophagy pathway. Excessive copper can trigger oxidative stress and damage to organelles such as mitochondria and endoplasmic reticulum, leading to intrinsic apoptosis. Reasonable use of the relationship between copper and apoptosis may provide new insights for cancer treatment. For instance, applying copper ions to selectively induce cell apoptosis in tumor cells might be a potential therapeutic strategy. However, copper-induced cell death may not be limited to apoptosis, and other forms of cell death may be involved.

### 4.2 Copper overload triggers necroptosis

Necroptosis is a form of regulated cell death (RCD) that resembles necrosis morphologically, characterized by disruption of plasma membrane integrity, cytoplasm becoming semi-transparent, organelle swelling, and cell volume enlargement. Mechanistically, necroptosis is similar to apoptosis, mediated by the necrosome complex formed by receptor interacting protein kinase 1 (RIPK1), receptor interacting protein kinase 3 (RIPK3), and mixed lineage kinase domain-like protein (MLKL) (140). The RIPK1/RIPK3/MLKL complex induces necroptosis through multiple mechanisms, including promoting mitochondrial autophagy, producing mitochondrial reactive oxygen species, and altering mitochondrial permeability, leading to mitochondrial dysfunction (141). Various exogenous substances, such as metallic compounds and nanomaterials, can induce necroptosis (142). Treatment with copper chloride induces changes in mitochondrial permeability, DNA damage, organelle swelling, and ultimately necroptosis in primary liver cells from rainbow trout. Inhibition of necroptosis can reverse copper ion-induced cell death, indicating that copper induces necroptosis through a ROS-dependent DNA damage pathway. Copper ions are the most abundant metal ions in the nucleus of cells, and after treatment with copper chloride, copper ions can enter the nucleus and bind to DNA, leading to DNA fragmentation (143). Chen et al. reported the regulatory effect of copper ions and copper compounds on inducing necroptosis in cells, which can selectively intervene in tumor cells. Copper-based nanomaterials assembled from copper compounds have been widely used to induce necroptosis in tumor cells. For instance, CuS-MnS2 nanomaterials can induce necroptosis in ovarian cancer cells through magnetic resonance imaging (144). CuS-NiS2 nanomaterials are commonly used for clinical imaging diagnosis. Combined with infrared light therapy, CuS-NiS2 can induce the production of reactive oxygen species in human gastric cancer cells, activate the RIPK1/RIPK3/MLKL complex, and induce necroptosis in tumor cells, significantly reducing the size of tumors in mouse models (145).

These studies suggest a close relationship between copper and necroptosis, indicating that copper can serve as a therapeutic target for inducing necroptosis in tumor cells and has potential application prospects in tumor treatment.

### 4.3 Copper overload triggers pyroptosis

Pyroptosis is a form of programmed cell death induced by inflammasomes that can clear damaged cells, but unlike apoptosis, it can trigger surrounding inflammatory reactions. In different cell pyroptosis processes caused by various factors, there is an activation of corresponding inflammasomes, such as damage or pathogen-related molecules through the NOD-like receptor family, pyrin domain-containing 3 (NLRP3) pathway, or bacterial infections through the NOD-like receptor family CARD domain containing 4 (NLRC4) pathway. Abnormal double-stranded DNA can activate absent in melanoma 2 (AIM2), which further activates caspase-1, cleaves pro-IL-1β and pro-IL-18, and forms membrane pores on the cell membrane through the N-terminus of perforin D, releasing mature cytokines such as IL-1β and IL-18 (146). Studies have found that copper ions can mediate macrophage pyroptosis through the classic NLRP3 inflammasome activation pathway and participate in the regulation of inflammatory reactions. Specific blockade of NLRP3-mediated pyroptosis occurs upon the removal of copper, which may be related to the downregulation of copper-dependent SOD1 activity (147). When mouse macrophages were treated with copper oxide nanoparticles (CuONPs), an increase in protein levels of NLRP3, caspase-1, IL-1β, and an increase in the release of IL-1β was observed. NLRP3 siRNA reduced the expression of caspase-1, p20, and IL-1β induced by CuONPs, thus alleviating immune damage mediated by macrophage pyroptosis caused by CuONPs. This phenomenon indicates that CuONPs may promote NLRP3-dependent pyroptosis of macrophages through the NF-kB pathway (148). In experiments with primary chicken liver cells treated with CuSO4 and N-acetylcysteine (NAC), it was found that Cu^2+^ upregulated caspase-1 protein expression, increased the levels of caspase-1, IL-1β, and IL-18 in the culture supernatant, and induced vacuolar changes in the cells. The use of NAC reduced the mRNA and protein changes caused by Cu^2+^ overloading, indicating that Cu^2+^ overload can lead to the generation of reactive oxygen species in liver cells, thereby causing pyroptosis. Using a caspase-1 inhibitor partially reversed Cu^2+^-induced cell death, suggesting that Cu^2+^ damage to liver tissue is related to pyroptosis (149). In experiments with primary mouse microglia treated with CuCl2 and lipopolysaccharides, it was found that the expression levels of NLRP3, active caspase-1, apoptosis-associated speck-like protein containing a CARD (ASC), and IL-1β proteins increased in a time-dependent manner. Excessive copper exposure activated NLRP3 in microglia, leading to pyroptosis and subsequent neuronal damage (150). Copper exposure induced endoplasmic reticulum stress-dependent pyroptosis in jejunal epithelial cells, exerting a toxic effect. Researchers found that high copper feeding led to decreased pig weight. Treatment of jejunal epithelial cells with CuCl2 resulted in decreased cell viability, increased cell rupture, and increased expression of endoplasmic reticulum stress- and pyroptosis-related genes. The use of the endoplasmic reticulum stress inhibitor MKC-3946 effectively inhibited the IRE1α-XBP1 signaling pathway, thereby reducing copper-induced pyroptosis. These results indicate that excessive copper intake may cause intestinal toxicity by inducing pyroptosis in intestinal epithelial cells (151). In a lung tissue simulation system constructed from differentiated THP-1 macrophages and human non-small cell lung cancer A549 cells, treatment with CuONPs resulted in upregulation of the chemokine C-C motif chemokine ligand 22 (CCL22) and IL-1β genes in A549 cells and increased superoxide dismutase 2 (SOD2) expression. When lung epithelial cells were exposed to CuONPs, they released chemokines to recruit macrophages to participate in the inflammatory response. Copper-induced NLRP3 inflammasome-dependent pyroptosis and activating pro-inflammatory transcription factors in macrophages further amplify and strengthen the feedback loop to increase the level of inflammation represented by IL-1β, suggesting that copper is related to the regulation of local immune function in the lungs (152).

The above findings suggest that copper plays a crucial role in inducing pyroptosis, indicating that copper could be an important therapeutic target for pyroptosis-related diseases.

### 4.4 Copper overload triggers ferroptosis

Ferroptosis is a form of programmed cell death dependent on iron ions and distinct from apoptosis, necrosis, and autophagy. Its main characteristic is the accumulation of disrupted iron homeostasis and lipid peroxidation. Changes in mitochondrial structure and morphology have also been observed, such as increased mitochondrial membrane density, decreased or disappeared mitochondrial cristae, and ruptured outer mitochondrial membranes (153). Ferroptosis is mainly related to iron, but other metals may also be involved in its occurrence (154). Copper ions are related to iron metabolisms, such as the copper-dependent CP-catalyzed oxidation of Fe^2+^ to Fe^3+^ or copper-iron interactions in the intestine. Excess Cu^+^/Cu^2+^ and Fe^2+^/Fe^3+^ promote the production of reactive hydroxyl radicals through the Fenton reaction, leading to permanent modifications of cellular lipids, nucleic acids, and proteins and causing oxidative damage and cell death (155). Current research indicates that copper ions are closely related to ferroptosis. Studies have found that copper can induce neuronal cell ferroptosis, and FeCl2 and CuCl2 can both deplete GSH in mouse hippocampal neurons while enhancing ferroptosis induced by erastin, sulfasalazine (SSZ), and buthionine sulfoximine (BSO). Cu^2+^ has a significantly better effect than Fe^2+^ in enhancing the toxicity of SSZ and erastin, such as elevating reactive oxygen species and reducing glutathione peroxidase (GPX) (156). Copper can induce male reproductive toxicity. Copper sulfate (CuSO4) significantly promotes autophagy levels in testicular and mouse germ cell line GC-1 spg cells by affecting the AMPK-mTOR pathway and induces ferroptosis while inhibiting autophagy can inhibit CuSO4-induced ferroptosis *in vivo* and *in vitro (* 157). Some scholars have pointed out that ferroptosis has excellent potential in cancer treatment (158). Copper can stimulate ferroptosis through oxidative stress, so copper ionophores or copper chelators developed using biological properties can be used as a new type of anti-tumor drug. For example, drugs such as elesclomol, DSF, and TTM can induce oxidative stress and ferroptosis by inhibiting the steady-state balance of iron and copper (129). Application of elesclomol to colon cancer cells significantly increased the level of Cu^2+^ in mitochondria, increased oxidative stress pressure within cells, and caused ferroptosis (154). Combined treatment with DSF and Cu^2+^ can inhibit the viability of nasopharyngeal carcinoma cells. Transcriptome analysis showed that the ferroptosis pathway is related to cell death induced by DSF/Cu, and seven genes related to ferroptosis showed significant changes. This phenomenon suggests that the ability of DSF/Cu to inhibit nasopharyngeal carcinoma cells may depend to some extent on ferroptosis (159). Another study found that DSF/Cu can disrupt the stability of liver cell mitochondria, causing mitochondrial fragmentation and accumulation around the cell nucleus. Combined application of DSF/Cu and sorafenib has a significant inhibitory effect on Huh7 cells. The content of free iron, superoxide, and lipid peroxides in the cells increased. Nude mouse transplantation tumor model experiments showed that the combined use of DSF and sorafenib showed a synergistic tumor growth inhibition effect, indicating that the combined use of copper and anti-tumor drugs has a synergistic promoting effect on ferroptosis of HCC cells (160).

The studies mentioned above suggest that regulating intracellular copper ion concentration can influence ferroptosis in cells. Therefore, targeting copper ions can be applied to enhance the sensitivity of tumor chemotherapy based on ferroptosis, which has important implications in cancer chemotherapy.

### 4.5 Copper overload triggers cuproptosis

Recently, Tsvetkov et al. published a critical study indicating that an excessive accumulation of copper ions in human cells can trigger a new form of cell death, distinct from known pathways such as apoptosis, ferroptosis, pyroptosis, and necroptosis. This copper-dependent form of cell death is known as “cuproptosis” (13). The occurrence of cuproptosis depends on the accumulation of copper within cells and is closely associated with mitochondrial respiration. Cells with active mitochondrial respiration are about 1000 times more sensitive to cuproptosis induced by disulfiram than those with active glycolysis (13). The researchers found that ferredoxin 1 (FDX1) and protein lipoylation are vital regulators of cuproptosis and confirmed that FDX1 is an upstream regulator of the lipoylated protein dihydrolipoamide S-acetyltransferase (DLAT). When extracellular Cu^2+^ enters the mitochondrial compartment of cells, FDX1 reduces Cu^2+^ to Cu^+^, resulting in the oligomerization of DLAT protein and the downregulation of Fe-S cluster protein expression. These events ultimately increase the level of toxic stress proteins of the heat shock protein family A (HSP70), inducing protein toxicity stress and cell death (13). Our prior investigations using bioinformatics analytics underscored a notable correlation between the cuproptosis phenotype and the prognosis of patients afflicted with HCC, insinuating a significant therapeutic potential of cuproptosis in oncology (161). In the burgeoning field of cancer therapy, cuproptosis has already begun to take shape as demonstrated by numerous research reports. A study by Huo, Shengqi et al., for instance, proposed cuproptosis as a prospective pathway to inhibit the advancement of colorectal cancer. They divulged that the chemical compound 4-Octyl itaconate (4-OI) amplifies the impact of cuproptosis by hindering aerobic glycolysis and directing its action towards GAPDH, a crucial enzyme in glycolysis. This sophisticated interplay of elesclomol-Cu and 4-OI has demonstrated successful tumor growth reduction in both *in vitro* and *in vivo* environments (162). Furthermore, when combined with nanomedicine, cuproptosis displays remarkable potential in cancer treatment. A groundbreaking approach introduced by Guo, Boda et al., outlines a novel cancer therapy tactic that involves the creation of a ROS-sensitive polymer, PHPM, which is utilized to concurrently encapsulate elesclomol and copper into nanoparticles, dubbed NP@ESCu. This delivery system efficiently transports Cu into the cancer cells, initiating cuproptosis and sparking immune responses. Notably, an alliance of NP@ESCu with anti-programmed cell death protein ligand-1 antibody (αPD-L1) has indicated favorable outcomes in mouse models, thereby illuminating a potentially novel direction for bolstered cancer treatment in the future (163).

Cuproptosis, a newly discovered form of cell death, has opened up new avenues for the treatment of copper metabolism-related diseases such as cancer. Further exploration of the potential mechanisms of cuproptosis in the development of tumors and the investigation of the related regulatory pathways of cuproptosis in different pathological backgrounds have significant research value and translational significance for the clinical treatment of cancer.

## 5 Potential tumor treatment strategies for copper

**Figure 3:**
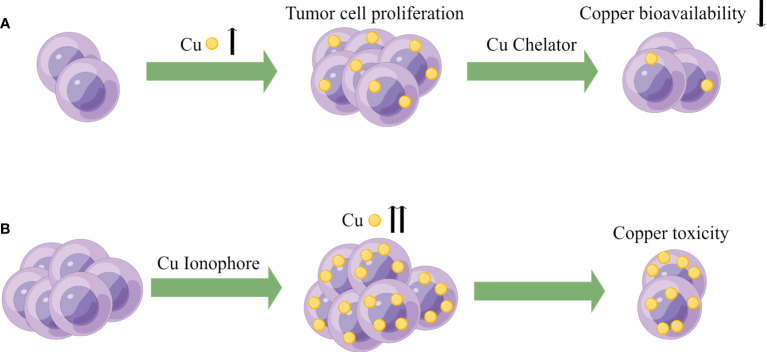
Potential oncology treatment strategies of copper. **(A)** Based on the higher demand for copper in tumor cells, copper bioavailability can be reduced using Cu chelators to achieve the goal of cancer therapy. **(B)** Exploiting the higher copper burden in tumor cells, Cu ionophores can be utilized to increase intracellular copper concentration, inducing copper toxicity to achieve the goal of cancer treatment.

Resistance to cell death is one of the most important characteristics of cancer cells and a major cause of treatment resistance (164). Targeting copper ions can induce cell death, and therefore, based on the enrichment of copper in cancer tissues, it can help distinguish cancer cells from healthy cells. Targeting copper has become one of the hotspots in the development of anti-cancer drugs (47, 165). Currently, there are two main strategies for targeting copper in cancer treatment. The first is copper chelators, such as D-penicillamine, TTM, and trientine. These drugs reduce the bioavailability of copper by binding with copper, thus achieving the goal of treating cancer. The second is copper ionophores, such as elesclomol and disulfiram. These carriers can increase the intracellular level of copper ions and exert anti-tumor effects by producing reactive oxygen species, inhibiting proteasomes, and inducing apoptosis (129, 166). The schematic diagram is shown in **Figure 3**. **Table 1** summarizes some FDA-approved and experimental drugs that target copper metabolism for the treatment of cancer.

**Table 1:** FDA approved and experimental drugs targeting copper metabolism in cancer treatment.

Drug Name	Mechanism	FDA Approval Status	Disease
D-penicillamine	Binds to copper and leads to its excretion	Yes	Wilson’s disease
Trientine	Binds to copper and leads to its excretion	Yes	Wilson’s disease
Zinc acetate	Blocks absorption of copper in the gut	Yes	Wilson’s disease
Tetrathiomolybdate	Binds to copper and leads to its excretion	No	N/A
Ethylenediaminetetraacetic acid (EDTA)	Binds to copper and leads to its excretion	Yes	Lead poisoning and iron overload
Dimercapto-propane sulfonate (DMPS)	Binds to copper and leads to its excretion	No	N/A
Dimercaptosuccinic acid (DMSA)	Binds to copper and leads to its excretion	Yes	Lead poisoning in children
Disulfiram (DSF)	Transports copper into cells and leads to its overload	Yes	Alcoholism
Elesclomol	Transports copper into cells and leads to its overload	No	N/A
Diacetyl-bis (N4-methylthiosemicarbazone) (ATSM)	Transports copper into cells and leads to its overload	No	N/A
Glyoxal-bis (N4-methylthiosemicarbazone) (GTSM)	Transports copper into cells and leads to its overload	No	N/A

N/A, Not applicable.

### 5.1 The therapeutic potential of decreasing copper bioavailability in tumor

In recent years, it has been increasingly recognized that compared to most other tissues, tumor cells have an increased demand for copper, which represents a metabolic vulnerability that can be exploited by limiting the availability of copper (167). Serum copper levels in HCC patients are significantly higher than in the chronic hepatitis control group (137 ± 24 vs 107 ± 15 μg/dl, *p* = 0.0030) (119, 168). Higher copper concentrations have also been found in the liver tissue of HCC dogs (169). Elevated serum copper levels are closely associated with poor liver cancer-specific survival and overall survival of patients (170). Therefore, some researchers have suggested that limiting the bioavailability of copper may be a targetable vulnerability in tumor therapy.

Metal chelators are compounds that selectively bind to specific atoms or ions (171). TTM is a copper chelator that can be used to treat HCC and reduce its proliferative capacity. Under hypoxic conditions, copper deficiency can decrease compensatory glycolysis in HCC cells, thereby weakening tumor characteristics under low oxygen conditions. This suggests that the pharmacological reduction of copper can inhibit the reprogramming of glucose metabolism in HCC (172). TTM can also suppress the development of spontaneous mammary tumors in mouse models, and tumor recurrence occurs several weeks after stopping TTM treatment, indicating that long-term copper deficiency keeps tumor cells in a dormant state (98, 173). Using TTM can target copper-dependent kinase activity of MEK1 and MEK2, inhibiting the initial and resistant forms of melanoma driven by B-Raf proto-oncogene, serine/threonine kinase (Braf) mutations in mice (174). In TTM clinical trials, serum ceruloplasmin levels were measured to monitor copper depletion and adjust drug dosage and treatment duration. In this study on breast cancer, 75 patients received two cycles of TTM treatment (48% with the most aggressive triple-negative breast cancer). The study lasted two years, and 51 patients completed treatment. The results showed that the decrease in serum ceruloplasmin levels in patients with severe copper depletion was associated with a reduction in circulating vascular endothelial growth factor receptor 2 (VEGFR2) and serum LOXL2, indicating that TTM treatment can affect the pre-metastatic niche of tumors. Patients tolerated the treatment well, with only lower levels of neutropenia (175). During MAPK pathway inhibition therapy for tumors, protective autophagy and ULK-dependent signaling pathways are often upregulated in KRAS and BRAF-mutant tumors. RAF-MEK-ERK signaling and copper-dependent autophagy driven by ULK1 and ULK2 make limiting copper bioavailability an effective strategy for blocking KRAS and BRAF-driven tumor growth and survival (176, 177). Cisplatin is an antitumor drug, and its uptake is mediated by copper transporter CTR1 inside the cell (178). Studies have found that low levels of CTR1 expression in human ovarian tumors are associated with poor responses to platinum-based therapy. In a human cervical cancer mouse model, combined treatment with a copper chelator and cisplatin increased the levels of cisplatin-DNA adducts in cancerous tissue without affecting normal tissue, thereby enhancing the therapeutic effect (178). Copper chelation therapy also has a promising mechanism involving programmed death-ligand 1 (PDL1). Cancer cells overexpress PDL1 to protect themselves from antitumor immune responses. Studies have shown a strong correlation between CTR1 and PDL1 expression in many tumors but not in normal tissues. Copper chelation can promote ubiquitin-mediated PDL1 degradation in the colon cancer cell line DLD1, while copper supplementation enhances PDL1 expression in tumor tissue. This phenomenon suggests that copper chelation therapy may synergize with antitumor immune therapy to enhance antitumor immune responses (119). Moreover, research has shown that mitochondrial copper depletion can cause metabolic reprogramming from oxidative metabolism to glycolysis, reducing energy production, which is an effective treatment method, particularly for cancers that depend on oxidative phosphorylation metabolism. Copper-depleting nanocrystals (CDNs) targeting mitochondrial copper are effective in treating triple-negative breast cancer (TNBC), inducing metabolic transformation from respiratory metabolism to glycolysis. CDN treatment can suppress tumor growth and increase the survival rate of three TNBC mouse models (179). Mitochondrial copper depletion can inhibit mitochondrial oxidative phosphorylation and target sex determining region Y-box 2/octamer-binding transcription factor 4 positive cells (SOX2/OCT4+ cells) with highly metastatic properties in TNBC by suppressing their metabolic reprogramming, which may potentially inhibit TNBC metastasis (180). In addition, clinical trials have shown that Copper chelators can be used as adjuvant therapy to improve the effectiveness of radiotherapy and chemotherapy in various types of cancer (60, 181). Serum copper levels are negatively correlated with the response to radiotherapy in cancer patients, indicating that serum copper is an effective monitoring indicator for radiotherapy efficacy (182). A recent study has demonstrated that an increase in Cu^2+^ levels in cells promotes radioresistance in HCC cell lines. Through cell experiments and mouse radiation models, the researchers found that the copper metabolism MURR1 domain 10 (COMM Domain Containing 10, COMMD10) decreased with increasing radiation dose. The loss of COMMD10 led to copper accumulation in cells, which then inhibited the ubiquitination and degradation of HIF-1α, promoted HIF-1α expression, and induced downstream solute carrier family 7 member 11 (SLC7A11) and CP overexpression. SLC7A11 can increase GSH synthesis and decrease lipid peroxidation levels. CP can decrease intracellular iron concentration, ultimately inhibiting HCC cell ferroptosis and weakening the efficacy of radiotherapy. This experiment suggests that copper homeostasis may be the weak point of radiotherapy resistance. By altering intracellular copper homeostasis and reducing intracellular copper levels, the radiosensitivity of HCC can be enhanced (183). In terms of drug safety, copper chelators have demonstrated good tolerability in treating some chronic genetic diseases, such as copper metabolism disorders (184). **Table 2** summarizes the crucial anticancer mechanisms associated with copper deficiency discussed above.

**Table 2:** Crucial anticancer mechanisms associated with copper deficiency and their detailed descriptions.

Mechanism	Detailed Description	Reference
Suppress Compensatory Glycolysis	Copper deficiency under hypoxia can inhibit compensatory glycolysis in HCC cells, thus reducing tumor characteristics.	(172)
Maintain Tumor Cells Dormancy	Long-term copper deficiency can keep tumor cells dormant, suppressing tumor recurrence.	(98, 173)
Target MEK1/2	By targeting copper-dependent kinase activity of MEK1/2, Braf-driven melanoma can be inhibited.	(174)
Improve Pre-Metastatic Niche	Copper deficiency, related to decreased VEGFR2 and LOXL2, suggests potential effects on the pre-metastatic tumor environment.	(175)
Synergize with MAPK Pathway Inhibitors	Copper deficiency can inhibit autophagy-related proteins ULK1/2, reducing protective autophagy induced by KRAS and BRAF mutations during MAPK pathway inhibition.	(176, 177)
Enhance Cisplatin Efficacy	Copper chelation reduces copper’s competitive binding with CTR1, enhancing cisplatin’s binding, transport, and efficacy.	(178)
Promote PDL1 Degradation	Copper chelation can promote ubiquitin-mediated degradation of PDL1 in tumor cells, potentially synergizing with anti-tumor immunotherapy.	(119)
Metabolic Reprogramming	Copper deficiency in mitochondria-dominant tumor cells can shift metabolism from oxidative to glycolytic, reducing energy production.	(179, 180)
Increase Radiosensitivity	Regulating intracellular copper ion homeostasis and reducing their levels can enhance the radiosensitivity of HCC.	(60, 181–183)

In conclusion, copper chelation therapy presents a promising avenue for cancer treatment due to its ability to exploit the increased demand for copper in tumor cells, affect cellular metabolic processes, and enhance the effectiveness of other cancer therapies. However, further studies and clinical trials are needed to fully understand its therapeutic potential and safety in various types of cancer.

### 5.2 Antitumor effects of copper accumulation induced in cells

On the other hand, using copper ionophores to increase intracellular copper ion concentration has also shown promising therapeutic effects in cancer treatment. Many different types of copper ionophores have been used as anticancer agents, including DSF, elesclomol, and others. As early as several decades ago, researchers recognized that cancer cells are more sensitive to elevated ROS levels compared to normal cells (185, 186). Therefore, despite copper’s promoting effects on cell survival and proliferation, and the dependence of tumor cells on copper, inducing copper accumulation in cancer cells can specifically induce the production of higher levels of ROS in tumor tissues based on the sensitivity of cancer cells to ROS and the higher concentration of copper ions in cancer tissues. This strategy may achieve the goal of treating tumors (187).

DSF is an FDA-approved aldehyde dehydrogenase inhibitor with a history of over 60 years in treating alcoholism. However, the bioactivity of DSF as a copper ionophore is increasingly gaining attention for rediscovering its potential as an anticancer drug (188, 189). Firstly, cancer cells contain more copper ions, which can react with DSF to form a complex of diethyldithiocarbamate-copper [(Cu (DDC)2 CuET)]. This complex has a specific targeting effect, selectively acting on tumor cells without affecting healthy cells. DSF can also inhibit SOD in cancer cells and compete with glutathione reductase to block the activity of aldehyde dehydrogenase isozymes, inducing oxidative stress and DNA damage (166). DSF can also induce apoptosis by inducing mitochondrial permeability transition through the activation of mitogen-activated protein kinase. Furthermore, the binding of DSF and copper can serve as an inhibitor for various functional proteinases related to the proteasome in multiple cancers, inducing the accumulation of essential proteins such as IKB, P27, and c-MYC, causing cell cycle arrest and triggering apoptosis (190). Furthermore, DSF-Cu complexes can significantly inhibit NF-kB activity, weaken the epithelial-mesenchymal transition of liver cancer cells, promote apoptosis, and increase sensitivity to antitumor drugs (191). DSF also has the characteristics of low cost and high safety (131). Ren et al. demonstrated that copper ionophore disulfiram (DSF) could selectively target HCC cells through iron death mechanisms, and the combination of DSF/Cu with sorafenib, which inhibits nuclear factor erythroid 2-related factor 2 (NRF2) function, may provide a promising synergistic strategy to overcome drug resistance and enhance tumor treatment (160). Many studies have shown that DSF-Cu preferentially targets cancer cells compared to normal cells and seems to preferentially target cancer stem cells (192–194). For example, Yip et al. found that DSF-Cu could inhibit breast cancer stem cells and enhance the effect of paclitaxel treatment. This phenomenon may be due to the induction of ROS generation and activation of downstream apoptosis-related pathways while inhibiting NFκB activity (195). Some other studies have shown that the combination of disulfiram and copper can induce tumor cell apoptosis by inhibiting proteasome activity (196, 197). This mechanism of inhibiting proteasome activity may be achieved by targeting nuclear protein localization protein 4 homolog (NPL4), which is the adapter factor for the p97 (also known as VCP) segregase, involved in the conversion of proteins involved in multiple regulatory and stress response pathways (198). Studies have also shown that disulfiram-copper (DSF-Cu) can inhibit aldehyde dehydrogenase (ALDH), targeting cancer stem cells, and therefore has a synergistic effect on the chemoresistance of tumor stem cells that affect sorafenib treatment outcomes (199). HCC cells treated with DSF/Cu show immunogenic cell death (ICD) characteristics *in vitro* and can enhance the effect of CD47 blockade therapy by immune activation (200). In recent years, researchers have been working to address the problems of the rapid metabolism, poor stability, and short half-life of DSF in order to more fully utilize its anti-tumor properties (201).

Elesclomol is another well-known copper ionophore that has recently been studied for various cancer treatments. Unlike DSF, elesclomol can directly transport copper ions to the mitochondria inside cells. Moreover, at the same concentration, elesclomol can significantly increase the level of copper ions inside cells compared to DSF (129). It has been reported that the use of elesclomol can decompose ATP7A in colon cancer cells, which is a protein that mediates the export of copper ions inside cells (202). The decomposition of ATP7A further leads to the accumulation of copper ions in the mitochondria of cancer cells (154). The mechanism of elesclomol for treating tumors is controversial. It was previously thought that its mechanism of action was to induce oxidative stress to cause apoptosis of cancer cells. It is well known that compared with normal cells, tumor cells have a higher baseline level of ROS, which helps to develop or maintain a malignant phenotype. At the same time, this also makes cancer cells more susceptible to irreversible oxidative damage and subsequent cell death (203, 204). Therefore, some researchers believe that elesclomol can increase ROS levels by inducing oxidative stress, exceeding the lethal threshold for cancer cells while maintaining normal cell vitality (132, 205). However, some studies suggest that the generation of ROS does not seem to be necessary for elesclomol to exert its cytotoxicity. Some studies attempted to reverse the induction of ROS by using the ROS scavenger NAC; the cytotoxicity of elesclomol can only be reversed by using NAC in some cells (206). Some studies have found that the cytotoxic effects of elesclomol on various small cell lung cancer (SCLC) cell lines, including SCLC 1, SCLC SR2, SCLC B, and SCLC BC (207) and non-small cell lung cancer cell line A549 (205), can be reversed by using 0.1 mM and 10 mM NAC. However, in GSC, 5 mM NAC does not help reverse the cytotoxicity of elesclomol, while 10 mM NAC only has a partial reversal effect (139). Another study also showed that 5 mM NAC only slightly mitigated the loss of activity in three cell lines, NCIH2030, A549, and HCC4009, in response to elesclomol (13). Therefore, the anti-cancer effect of elesclomol is partially related to its induction of ROS, but there should be more critical mechanisms to explain its cancer cell toxicity. Some scholars have pointed out that the differences between normal cell mitochondria and cancer cell mitochondria (such as membrane permeability, reactions to uncoupling, and the structure and function of complex I) may somehow contribute to the selective cytotoxicity of elesclomol towards certain cancer cell types. In fact, scientific research over the past century has revealed many significant differences in mitochondrial structure and function between normal cells and cancer cells, including differences in mtDNA sequences, molecular composition, and metabolic activity (208). Subsequent studies have found that elesclomol-induced cell damage also involves DNA damage and cell cycle arrest (209). In addition, it has been reported that elesclomol can induce ferroptosis (154). However, whichever mechanism is involved is related to elesclomol’s copper ion transport function.

The study conducted by Nagai, Masazumi et al. indicated that elesclomol could chelate extracellular copper at very low concentrations (nM levels) and enter cells in the form of elesclomol-Cu^2+^ complex, selectively targeting the mitochondria and the mitochondrial enzyme FDX1. FDX1 can reduce Cu^2+^ to Cu^+^, resulting in the generation of a large number of reactive oxygen species that trigger apoptosis in cancer cells, exhibiting anti-tumor activity against various cancer cells (129). However, like previous studies, researchers attributed the cytotoxicity of elesclomol to the excessive production of ROS and could not explain why the cell death induced by elesclomol was closely related to mitochondrial respiration and why the sensitivity of cells with active mitochondrial respiration to elesclomol was nearly 1000 times higher than that of cells with active glycolysis. Recently, Tsvetkov, Peter et al. proposed the concept of “cuproptosis” as a unique mechanism to explain the cytotoxic effect of elesclomol. The researchers found that FDX1 and protein lipoylation were key regulatory factors for cuproptosis and confirmed that FDX1 was an upstream regulatory factor of the lipoylated protein DLAT. When extracellular Cu^2+^ entered the mitochondria, FDX1 reduced Cu^2+^ to Cu^+^, leading to oligomerization of DLAT protein, a decrease in the expression of Fe-S cluster proteins, and an increase in the level of toxic stress protein in the heat shock protein family A (HSP70), thereby inducing protein toxicity stress and cell death [6]. DLAT is part of the respiratory chain complex II (cytochrome c reductase) and mainly catalyzes the generation of acetyl-CoA in the pyruvate dehydrogenase (PDH) reaction, which is further oxidized in the TCA cycle. Therefore, cuproptosis markers such as FDX1 and DLAT are significantly correlated with mitochondrial respiration intensity. Tsvetkov, Peter et al. also found that the occurrence of cuproptosis was significantly related to mitochondrial metabolism, providing a more plausible mechanism for explaining the anti-cancer effect of ES depending on the transport of extracellular copper ions and the mitochondrial respiration intensity of cancer cells (13).

The metabolic selectivity of elesclomol may provide a reference for the treatment of specific metabolic-type tumors as a targeted therapy (210). For example, targeting drug-resistant cancer cells, many drug-resistant cancer cells exhibit vigorous mitochondrial respiration activity, including drug-resistant cancer cells to cisplatin and proteasome inhibitors. The ID 50 of elesclomol in cisplatin-resistant cells is in the low nanomolar range (207). Furthermore, cancer stem cells and molecularly targeted drug-resistant cancer cells also show significantly enhanced mitochondrial metabolism. As elesclomol displays substantial toxicity against all cells with strong mitochondrial respiration, it could be a feasible strategy to use elesclomol as an adjuvant for chemotherapy in combination with other drugs. Chemotherapy drugs can force cancer cells to selectively alter their metabolic patterns, increasing their dependence on mitochondrial metabolism to adapt to drug-induced changes. However, it should be noted that cells with high mitochondrial metabolism may be sensitive to the effects of elesclomol (132). Under the dual pressure of chemotherapy drugs and elesclomol, cancer cells face opposing pressures, which may help prevent the development of drug resistance. In addition, a combination of glycolysis inhibitors and elesclomol might be an effective solution. Although there are currently no reports of combined treatment with elesclomol and glycolysis inhibitors for cancer, related investigations predict that combining these two treatments will yield sound therapeutic effects. In preclinical models, the combination of elesclomol and pyruvate dehydrogenase kinase (PDK) inhibitor DCA has shown promising results. DCA is a small molecule that shifts cell metabolism from glycolysis to mitochondrial metabolism (211). The metabolic plasticity of cancer cells increases their dependence on mitochondrial metabolism in response to glycolysis inhibition, which promotes the function of elesclomol. In clinical trials, elesclomol combined with paclitaxel has been evaluated in multiple major clinical trials targeting advanced melanoma (212–214). A Phase I clinical trial showed that the combination of elesclomol and paclitaxel had good tolerability, with toxicity characteristics similar to paclitaxel alone (214). A Phase II study showed that adding elesclomol to paclitaxel increased the median progression-free survival by double, reduced disease progression/death risk by 41.7%, and improved overall survival (212). Although the results of a Phase III clinical trial showed that using elesclomol to treat melanoma did not significantly benefit tumor patients, elesclomol had anti-tumor effects on patients with low plasma lactate dehydrogenase levels. As the increase in lactate dehydrogenase activity is related to tumor hypoxia, this finding is consistent with the fact that elesclomol is more sensitive to tumor cells that depend on mitochondrial respiration (213).

Elesclomol has also shown good performance in terms of drug safety, with some studies reporting that elesclomol’s cytotoxicity appears to be selective for cancer cells (129). Human peripheral blood mononuclear cells (PBMCs) are unaffected at concentrations of elesclomol that have a significant killing effect on cancer cells, and elesclomol cannot induce copper ion enrichment in PBMCs (212). Clinical trial results have also not reported severe side effects from the use of elesclomol alone or in combination with other chemotherapy agents. According to existing reports, nearly a thousand patients have received high doses of elesclomol in clinical trials. The excellent tolerance of patients to elesclomol is a common feature of these trials. For example, in a Phase I trial, the maximum tolerated dose of elesclomol in solid tumor patients was as high as 438 mg/m^2^ (214). In past clinical trials, no patients reported organ or functional damage associated with elesclomol (212, 214, 215). Therefore, elesclomol treatment has a high level of safety. **Table 3** summarizes the primary anticancer mechanisms related to copper accumulation discussed above.

**Table 3:** Primary anticancer mechanisms related to copper accumulation and their detailed descriptions.

Mechanism	Detailed Description	Reference
Induce ROS Production	As cancer cells are more sensitive to ROS, copper ionophore induce high ROS levels, causing toxic damage and death in cancer cells.	(129, 132, 185–187, 203–205)
Specific Targeting Effect	Copper ionophore like DSF form Cu (DDC)2 CuET complexes, which selectively target tumor cells without affecting healthy cells	(129, 190–194, 212)
Induce DNA Damage and Cell Cycle Arrest	Copper ionophore can induce DNA damage, leading to cell cycle arrest, thus inhibiting tumor growth.	(209)
Induce Cell Death	Copper ionophore can induce cancer cell death via several mechanisms. For example, elesclomol can induce cell death through ferroptosis and cuproptosis.	(13, 154, 160)
Inhibit Tumor-Related Enzyme Activity	Copper ionophore like DSF can inhibit superoxide dismutase (SOD) in tumor cells, compete with glutathione reductase, prevent the activity of aldehyde dehydrogenase isoenzymes, leading to oxidative stress and DNA damage.	(160, 166, 191)
Inhibit Proteasome Activity	DSF and Elesclomol can inhibit various functional proteases related to the proteasome, causing accumulation of essential proteins (like IKB, P27, and c-MYC), thus inducing cell cycle arrest and apoptosis.	(190, 196–198)
Target Cancer Stem Cells	DSF and Elesclomol can target cancer stem cells, enhancing the sensitivity and anti-tumor effects of anti-cancer drugs.	(195, 199)
Promote Immune Activation	DSF can enhance the effect of CD47 blockade therapy by inducing ICD.	(200)
Selective Toxicity to Specific Metabolic Type Tumors	Elesclomol shows significant toxicity to cells with high mitochondrial respiration activity, including drug-resistant cancer cells, cancer stem cells, and cancer cells resistant to molecular targeted drugs.	(132, 207, 210, 211, 213)

In conclusion, the discovery of copper-mediated apoptosis has further refined the specific anti-cancer mechanism of elesclomol. Progress in studying the relationship between elesclomol and mitochondrial metabolism and copper-induced apoptosis provides the possibility of exploring the reapplication of elesclomol in clinical settings. New clinical trials should selectively target cancer types with high mitochondrial metabolism and attempt to combine elesclomol with platinum agents, proteasome inhibitors, molecular targeted drugs or glycolysis inhibitors (216).

## 6 Future perspectives and outlook

Copper is one of the fundamentals for maintaining cellular physiological functions and is closely related to the proliferation, metastasis, angiogenesis, immune microenvironment, and resistance to radiotherapy and chemotherapy of tumor cells. The disruption of copper metabolism may mediate cytotoxic damage and induce tumor cell death, providing new strategies for cancer treatment. Therefore, further research on copper homeostasis and its regulatory mechanisms is essential. In addition, the role of lipoylation in copper-dependent cell death should be actively explored to develop potential applications for targeted intervention based on cuproptosis. Currently, various tumor types and subtypes associated with copper dependence or sensitivity have been proposed based on numerous correlation analyses, but further screening is required to develop personalized treatment plans.

Multiple copper chelators and copper ionophore have exhibited good anti-tumor properties, indicating that targeting the copper transport system can be used for tumor treatment or increasing the sensitivity of other anticancer drugs. In addition, enhancing mitochondrial metabolism by altering the metabolic status of tumor cells and using elesclomol specificity for mitochondrial respiration may also become applicable to cuproptosis therapy. Studies have already shown that metabolic reprogramming can enhance the efficacy of cuproptosis therapy on tumors. For example, Yang et al. used 4-octyl itaconate to inhibit aerobic glycolysis, thereby enhancing the effect of elesclomol-induced cuproptosis in colorectal cancer cells (162). Therefore, targeting copper metabolism homeostasis and inducing different forms of cell death, such as cuproptosis, holds enormous promise for the field of tumor treatment.

## Author contributions

XT, ZY, and YM contributed to the conceptualization of the study; XT, WH, and ZL performed the methodology; XT, ZY, and YM wrote the writing-original draft; DM and LY contributed significantly to the writing-review and editing. All authors contributed to the article and approved the submitted version.

## References

[1] Trumbo P Yates AA Schlicker S Poos M. Dietary reference intakes: vitamin a, vitamin K, arsenic, boron, chromium, copper, iodine, iron, manganese, molybdenum, nickel, silicon, vanadium, and zinc. J Am Diet Assoc (2001) 101:294–301. doi: 10.1016/S0002-8223(01)00078-5 11269606

[2] Tsang T Davis CI Brady DC. Copper biology. Curr Biol (2021) 31:R421–7. doi: 10.1016/j.cub.2021.03.054 33974864

[3] Arredondo M Núñez MT. Iron and copper metabolism. Mol Aspects Med (2005) 26:313–27. doi: 10.1016/j.mam.2005.07.010 16112186

[4] Yoshida Y Furuta S Niki E. Effects of metal chelating agents on the oxidation of lipids induced by copper and iron. Biochim Biophys Acta (1993) 1210:81–8. doi: 10.1016/0005-2760(93)90052-b 8257723

[5] Juarez JC Betancourt O Pirie-Shepherd SR Guan X Price ML Shaw DE. Copper binding by tetrathiomolybdate attenuates angiogenesis and tumor cell proliferation through the inhibition of superoxide dismutase 1. Clin Cancer Res (2006) 12:4974–82. doi: 10.1158/1078-0432.CCR-06-0171 16914587

[6] Yoshida D Ikeda Y Nakazawa S. Copper chelation inhibits tumor angiogenesis in the experimental 9L gliosarcoma model. Neurosurgery (1995) 37:287–92. doi: 10.1227/00006123-199508000-00014 7477781

[7] Kamiya T. Copper in the tumor microenvironment and tumor metastasis. J Clin Biochem Nutr (2022) 71:22–8. doi: 10.3164/jcbn.22-9 PMC9309082 35903604

[8] Lopez J Ramchandani D Vahdat L. Copper depletion as a therapeutic strategy in cancer. Met Ions Life Sci (2019) 19:303–30. doi: 10.1515/9783110527872-018 30855113

[9] Chen S-Y Liu S-T Lin W-R Lin C-K Huang S-M. The mechanisms underlying the cytotoxic effects of copper Via differentiated embryonic chondrocyte gene 1. Int J Mol Sci (2019) 20:5225. doi: 10.3390/ijms20205225 31652494 PMC6834119

[10] Gupte A Mumper RJ. Elevated copper and oxidative stress in cancer cells as a target for cancer treatment. Cancer Treat Rev (2009) 35:32–46. doi: 10.1016/j.ctrv.2008.07.004 18774652

[11] Jiang Y Huo Z Qi X Zuo T Wu Z. Copper-induced tumor cell death mechanisms and antitumor theragnostic applications of copper complexes. Nanomedicine (Lond) (2022) 17:303–24. doi: 10.2217/nnm-2021-0374 35060391

[12] Macomber L Imlay JA. The iron-sulfur clusters of dehydratases are primary intracellular targets of copper toxicity. Proc Natl Acad Sci U.S.A. (2009) 106:8344–9. doi: 10.1073/pnas.0812808106 PMC2688863 19416816

[13] Tsvetkov P Coy S Petrova B Dreishpoon M Verma A Abdusamad M. Copper induces cell death by targeting lipoylated TCA cycle proteins. Science (2022) 375:1254–61. doi: 10.1126/science.abf0529 PMC9273333 35298263

[14] Turnlund JR Keyes WR Anderson HL Acord LL. Copper absorption and retention in young men at three levels of dietary copper by use of the stable isotope 65Cu. Am J Clin Nutr (1989) 49:870–8. doi: 10.1093/ajcn/49.5.870 2718922

[15] Shanbhag VC Gudekar N Jasmer K Papageorgiou C Singh K Petris MJ. Copper metabolism as a unique vulnerability in cancer. Biochim Biophys Acta Mol Cell Res (2021) 1868:118893. doi: 10.1016/j.bbamcr.2020.118893 33091507 PMC7779655

[16] Bertini I Rosato A. Menkes disease. Cell Mol Life Sci (2008) 65:89–91. doi: 10.1007/s00018-007-7439-6 17989919 PMC11131655

[17] Poujois A Woimant F. Wilson’s disease: a 2017 update. Clin Res Hepatol Gastroenterol (2018) 42:512–20. doi: 10.1016/j.clinre.2018.03.007 29625923

[18] Chen L Min J Wang F. Copper homeostasis and cuproptosis in health and disease. Signal Transduct Target Ther (2022) 7:378. doi: 10.1038/s41392-022-01229-y 36414625 PMC9681860

[19] Kim B-E Nevitt T Thiele DJ. Mechanisms for copper acquisition, distribution and regulation. Nat Chem Biol (2008) 4:176–85. doi: 10.1038/nchembio.72 18277979

[20] Shi H Jiang Y Yang Y Peng Y Li C. Copper metabolism in saccharomyces cerevisiae: an update. Biometals (2021) 34:3–14. doi: 10.1007/s10534-020-00264-y 33128172

[21] Grubman A White AR. Copper as a key regulator of cell signalling pathways. Expert Rev Mol Med (2014) 16:e11. doi: 10.1017/erm.2014.11 24849048

[22] Ding X Xie H Kang YJ. The significance of copper chelators in clinical and experimental application. J Nutr Biochem (2011) 22:301–10. doi: 10.1016/j.jnutbio.2010.06.010 21109416

[23] Blockhuys S Celauro E Hildesjö C Feizi A Stål O Fierro-González JC. Defining the human copper proteome and analysis of its expression variation in cancers. Metallomics (2017) 9:112–23. doi: 10.1039/c6mt00202a 27942658

[24] Uauy R Olivares M Gonzalez M. Essentiality of copper in humans. Am J Clin Nutr (1998) 67:952S–9S. doi: 10.1093/ajcn/67.5.952S 9587135

[25] Turski ML Thiele DJ. New roles for copper metabolism in cell proliferation, signaling, and disease. J Biol Chem (2009) 284:717–21. doi: 10.1074/jbc.R800055200 PMC2613604 18757361

[26] Schägger H Pfeiffer K. Supercomplexes in the respiratory chains of yeast and mammalian mitochondria. EMBO J (2000) 19:1777–83. doi: 10.1093/emboj/19.8.1777 PMC302020 10775262

[27] Boekema EJ Braun H-P. Supramolecular structure of the mitochondrial oxidative phosphorylation system. J Biol Chem (2007) 282:1–4. doi: 10.1074/jbc.R600031200 17102127

[28] Hüttemann M Lee I Samavati L Yu H Doan JW. Regulation of mitochondrial oxidative phosphorylation through cell signaling. Biochim Biophys Acta (2007) 1773:1701–20. doi: 10.1016/j.bbamcr.2007.10.001 18240421

[29] McCord JM Fridovich I. Superoxide dismutase. an enzymic function for erythrocuprein (hemocuprein). J Biol Chem (1969) 244:6049–55. doi: 10.1016/S0021-9258(18)63504-5 5389100

[30] Yonashiro R Sugiura A Miyachi M Fukuda T Matsushita N Inatome R. Mitochondrial ubiquitin ligase MITOL ubiquitinates mutant SOD1 and attenuates mutant SOD1-induced reactive oxygen species generation. Mol Biol Cell (2009) 20:4524–30. doi: 10.1091/mbc.e09-02-0112 PMC2770940 19741096

[31] Lill R Freibert S-A. Mechanisms of mitochondrial iron-sulfur protein biogenesis. Annu Rev Biochem (2020) 89:471–99. doi: 10.1146/annurev-biochem-013118-111540 31935115

[32] Xu W Barrientos T Andrews NC. Iron and copper in mitochondrial diseases. Cell Metab (2013) 17:319–28. doi: 10.1016/j.cmet.2013.02.004 PMC3594794 23473029

[33] Vallières C Holland SL Avery SV. Mitochondrial ferredoxin determines vulnerability of cells to copper excess. Cell Chem Biol (2017) 24:1228–1237.e3. doi: 10.1016/j.chembiol.2017.08.005 28867595 PMC5654725

[34] Desler C Hansen TL Frederiksen JB Marcker ML Singh KK Juel Rasmussen L. Is there a link between mitochondrial reserve respiratory capacity and aging? J Aging Res (2012) 2012:192503. doi: 10.1155/2012/192503 22720157 PMC3375017

[35] Dallman PR Goodman JR. Enlargement of mitochondrial compartment in iron and copper deficiency. Blood (1970) 35:496–505. doi: 10.1182/blood.V35.4.496.496 4315322

[36] Ruiz LM Jensen EL Bustos RI Argüelloa G Gutierrez-Garcia R González M. Adaptive responses of mitochondria to mild copper deprivation involve changes in morphology, OXPHOS remodeling and bioenergetics. J Cell Physiol (2014) 229:607–19. doi: 10.1002/jcp.24484 24446197

[37] Zhang L Chang CJ Bacus SS Hung MC. Suppressed transformation and induced differentiation of HER-2/neu-overexpressing breast cancer cells by emodin. Cancer Res (1995) 55:3890–6. 7543819

[38] Jensen EL Gonzalez-Ibanez AM Mendoza P Ruiz LM Riedel CA Simon F. Copper deficiency-induced anemia is caused by a mitochondrial metabolic reprograming in erythropoietic cells. Metallomics (2019) 11:282–90. doi: 10.1039/c8mt00224j 30358789

[39] dos Santos NV Matias AC Higa GSV Kihara AH Cerchiaro G. Copper uptake in mammary epithelial cells activates cyclins and triggers antioxidant response. Oxid Med Cell Longev (2015) 2015:162876. doi: 10.1155/2015/162876 26583055 PMC4637100

[40] Itoh S Kim HW Nakagawa O Ozumi K Lessner SM Aoki H. Novel role of antioxidant-1 (Atox1) as a copper-dependent transcription factor involved in cell proliferation. J Biol Chem (2008) 283:9157–67. doi: 10.1074/jbc.M709463200 PMC2431038 18245776

[41] Grochowski C Blicharska E Baj J Mierzwińska A Brzozowska K Forma A. Serum iron, magnesium, copper, and manganese levels in alcoholism: a systematic review. Molecules (2019) 24:1361. doi: 10.3390/molecules24071361 30959950 PMC6480471

[42] Feng Y Zeng J-W Ma Q Zhang S Tang J Feng J-F. Serum copper and zinc levels in breast cancer: a meta-analysis. J Trace Elem Med Biol (2020) 62:126629. doi: 10.1016/j.jtemb.2020.126629 32745979

[43] Galler T Lebrun V Raibaut L Faller P Wezynfeld NE. How trimerization of CTR1 n-terminal model peptides tunes Cu-binding and redox-chemistry. Chem Commun (Camb) (2020) 56:12194–7. doi: 10.1039/d0cc04693k 32914794

[44] Altarelli M Ben-Hamouda N Schneider A Berger MM. Copper deficiency: causes, manifestations, and treatment. Nutr Clin Pract (2019) 34:504–13. doi: 10.1002/ncp.10328 31209935

[45] Hansen SL Schlegel P Legleiter LR Lloyd KE Spears JW. Bioavailability of copper from copper glycinate in steers fed high dietary sulfur and molybdenum. J Anim Sci (2008) 86:173–9. doi: 10.2527/jas.2006-814 17911232

[46] Wang Y Hodgkinson V Zhu S Weisman GA Petris MJ. Advances in the understanding of mammalian copper transporters. Adv Nutr (2011) 2:129–37. doi: 10.3945/an.110.000273 PMC3065767 22332042

[47] Li Y. Copper homeostasis: emerging target for cancer treatment. IUBMB Life (2020) 72:1900–8. doi: 10.1002/iub.2341 32599675

[48] Saporito-Magriñá CM Musacco-Sebio RN Andrieux G Kook L Orrego MT Tuttolomondo MV. Copper-induced cell death and the protective role of glutathione: the implication of impaired protein folding rather than oxidative stress. Metallomics (2018) 10:1743–54. doi: 10.1039/c8mt00182k 30311620

[49] Harvey LJ Ashton K Hooper L Casgrain A Fairweather-Tait SJ. Methods of assessment of copper status in humans: a systematic review. Am J Clin Nutr (2009) 89:2009S–24S. doi: 10.3945/ajcn.2009.27230E 19420093

[50] Baldari S Di Rocco G Toietta G. Current biomedical use of copper chelation therapy. Int J Mol Sci (2020) 21:1069. doi: 10.3390/ijms21031069 32041110 PMC7037088

[51] Takahashi Y Kako K Kashiwabara S-I Takehara A Inada Y Arai H. Mammalian copper chaperone Cox17p has an essential role in activation of cytochrome c oxidase and embryonic development. Mol Cell Biol (2002) 22:7614–21. doi: 10.1128/MCB.22.21.7614-7621.2002 PMC135665 12370308

[52] Glerum DM Shtanko A Tzagoloff A. Characterization of COX17, a yeast gene involved in copper metabolism and assembly of cytochrome oxidase. J Biol Chem (1996) 271:14504–9. doi: 10.1074/jbc.271.24.14504 8662933

[53] Ge EJ Bush AI Casini A Cobine PA Cross JR DeNicola GM. Connecting copper and cancer: from transition metal signalling to metalloplasia. Nat Rev Cancer (2022) 22:102–13. doi: 10.1038/s41568-021-00417-2 PMC8810673 34764459

[54] Uriu-Adams JY Keen CL. Copper, oxidative stress, and human health. Mol Aspects Med (2005) 26:268–98. doi: 10.1016/j.mam.2005.07.015 16112185

[55] Festa RA Thiele DJ. Copper: an essential metal in biology. Curr Biol (2011) 21:R877–883. doi: 10.1016/j.cub.2011.09.040 PMC3718004 22075424

[56] Gudekar N Shanbhag V Wang Y Ralle M Weisman GA Petris MJ. Metallothioneins regulate ATP7A trafficking and control cell viability during copper deficiency and excess. Sci Rep (2020) 10:7856. doi: 10.1038/s41598-020-64521-3 32398691 PMC7217913

[57] Jiang X Chen J Bajić A Zhang C Song X Carroll SL. Quantitative real-time imaging of glutathione. Nat Commun (2017) 8:16087. doi: 10.1038/ncomms16087 28703127 PMC5511354

[58] Maryon EB Molloy SA Kaplan JH. Cellular glutathione plays a key role in copper uptake mediated by human copper transporter 1. Am J Physiol Cell Physiol (2013) 304:C768–779. doi: 10.1152/ajpcell.00417.2012 PMC3625801 23426973

[59] Kaplan JH Maryon EB. How mammalian cells acquire copper: an essential but potentially toxic metal. Biophys J (2016) 110:7–13. doi: 10.1016/j.bpj.2015.11.025 26745404 PMC4805867

[60] Denoyer D Masaldan S La Fontaine S Cater MA. Targeting copper in cancer therapy: “Copper that cancer”. Metallomics (2015) 7:1459–76. doi: 10.1039/c5mt00149h 26313539

[61] Zimnicka AM Tang H Guo Q Kuhr FK Oh M-J Wan J. Upregulated copper transporters in hypoxia-induced pulmonary hypertension. PloS One (2014) 9:e90544. doi: 10.1371/journal.pone.0090544 24614111 PMC3948681

[62] Su Y Zhang X Li S Xie W Guo J. Emerging roles of the copper-CTR1 axis in tumorigenesis. Mol Cancer Res (2022) 20:1339–53. doi: 10.1158/1541-7786.MCR-22-0056 35604085

[63] Peña MMO Lee J Thiele DJ. A delicate balance: homeostatic control of copper uptake and Distribution1. J Nutr (1999) 129:1251–60. doi: 10.1093/jn/129.7.1251 10395584

[64] Ishida S Andreux P Poitry-Yamate C Auwerx J Hanahan D. Bioavailable copper modulates oxidative phosphorylation and growth of tumors. Proc Natl Acad Sci U.S.A. (2013) 110:19507–12. doi: 10.1073/pnas.1318431110 PMC3845132 24218578

[65] Wooton-Kee CR Robertson M Zhou Y Dong B Sun Z Kim KH. Metabolic dysregulation in the Atp7b-/- wilson’s disease mouse model. Proc Natl Acad Sci U.S.A. (2020) 117:2076–83. doi: 10.1073/pnas.1914267117 PMC6994990 31924743

[66] Yang H Ralle M Wolfgang MJ Dhawan N Burkhead JL Rodriguez S. Copper-dependent amino oxidase 3 governs selection of metabolic fuels in adipocytes. PloS Biol (2018) 16:e2006519. doi: 10.1371/journal.pbio.2006519 30199530 PMC6130853

[67] Gunjan D Shalimar n Nadda N Kedia S Nayak B Paul SB. Hepatocellular carcinoma: an unusual complication of longstanding Wilson disease. J Clin Exp Hepatol (2017) 7:152–4. doi: 10.1016/j.jceh.2016.09.012 PMC5478940 28663680

[68] Atakul T Altinkaya SO Abas BI Yenisey C. Serum copper and zinc levels in patients with endometrial cancer. Biol Trace Elem Res (2020) 195:46–54. doi: 10.1007/s12011-019-01844-x 31399869

[69] Li Z Wang W Liu H Li S Zhang D. The association of serum zinc and copper with hypertension: a meta-analysis. J Trace Elem Med Biol (2019) 53:41–8. doi: 10.1016/j.jtemb.2019.01.018 30910205

[70] Zhang M Shi M Zhao Y. Association between serum copper levels and cervical cancer risk: a meta-analysis. Biosci Rep (2018) 38:BSR20180161. doi: 10.1042/BSR20180161 29519960 PMC6435553

[71] National Research Council (US) Committee on Copper in Drinking Water. Copper in drinking water (2000). Washington (DC: National Academies Press (US. Available at: http://www.ncbi.nlm.nih.gov/books/NBK225397/ (Accessed February 20, 2023).

[72] Fang A-P Chen P-Y Wang X-Y Liu Z-Y Zhang D-M Luo Y. Serum copper and zinc levels at diagnosis and hepatocellular carcinoma survival in the guangdong liver cancer cohort. Int J Cancer (2019) 144:2823–32. doi: 10.1002/ijc.31991 30426509

[73] Gupta SK Shukla VK Vaidya MP Roy SK Gupta S. Serum and tissue trace elements in colorectal cancer. J Surg Oncol (1993) 52:172–5. doi: 10.1002/jso.2930520311 8441275

[74] Zhang X Yang Q. Association between serum copper levels and lung cancer risk: a meta-analysis. J Int Med Res (2018) 46:4863–73. doi: 10.1177/0300060518798507 PMC6300955 30296873

[75] Dabek JT Hyvönen-Dabek M Härkönen M Adlercreutz H. Evidence for increased non-ceruloplasmin copper in early-stage human breast cancer serum. Nutr Cancer (1992) 17:195–201. doi: 10.1080/01635589209514187 1584712

[76] Barresi V Trovato-Salinaro A Spampinato G Musso N Castorina S Rizzarelli E. Transcriptome analysis of copper homeostasis genes reveals coordinated upregulation of SLC31A1, SCO1, and COX11 in colorectal cancer. FEBS Open Bio (2016) 6:794–806. doi: 10.1002/2211-5463.12060 PMC4971835 27516958

[77] Nagaraja GM Othman M Fox BP Alsaber R Pellegrino CM Zeng Y. Gene expression signatures and biomarkers of noninvasive and invasive breast cancer cells: comprehensive profiles by representational difference analysis, microarrays and proteomics. Oncogene (2006) 25:2328–38. doi: 10.1038/sj.onc.1209265 16314837

[78] Kastenhuber ER Lowe SW. Putting p53 in context. Cell (2017) 170:1062–78. doi: 10.1016/j.cell.2017.08.028 PMC5743327 28886379

[79] Loh SN. The missing zinc: p53 misfolding and cancer. Metallomics (2010) 2:442–9. doi: 10.1039/c003915b 21072344

[80] Formigari A Gregianin E Irato P. The effect of zinc and the role of p53 in copper-induced cellular stress responses. J Appl Toxicol (2013) 33:527–36. doi: 10.1002/jat.2854 23401182

[81] Porcu C Antonucci L Barbaro B Illi B Nasi S Martini M. Copper/MYC/CTR1 interplay: a dangerous relationship in hepatocellular carcinoma. Oncotarget (2018) 9:9325–43. doi: 10.18632/oncotarget.24282 PMC5823635 29507693

[82] Hwang JJ Park M-H Koh J-Y. Copper activates TrkB in cortical neurons in a metalloproteinase-dependent manner. J Neurosci Res (2007) 85:2160–6. doi: 10.1002/jnr.21350 17520746

[83] Michniewicz F Saletta F Rouaen JRC Hewavisenti RV Mercatelli D Cirillo G. Copper: an intracellular achilles’ heel allowing the targeting of epigenetics, kinase pathways, and cell metabolism in cancer therapeutics. ChemMedChem (2021) 16:2315–29. doi: 10.1002/cmdc.202100172 33890721

[84] He F Chang C Liu B Li Z Li H Cai N. Copper (II) ions activate ligand-independent receptor tyrosine kinase (RTK) signaling pathway. BioMed Res Int (2019) 2019:4158415. doi: 10.1155/2019/4158415 31218225 PMC6537018

[85] Turski ML Brady DC Kim HJ Kim B-E Nose Y Counter CM. A novel role for copper in ras/mitogen-activated protein kinase signaling. Mol Cell Biol (2012) 32:1284–95. doi: 10.1128/MCB.05722-11 PMC3302449 22290441

[86] Skrajnowska D Bobrowska-Korczak B Tokarz A Bialek S Jezierska E Makowska J. Copper and resveratrol attenuates serum catalase, glutathione peroxidase, and element values in rats with DMBA-induced mammary carcinogenesis. Biol Trace Elem Res (2013) 156:271–8. doi: 10.1007/s12011-013-9854-x PMC3844146 24213724

[87] Wang J Luo C Shan C You Q Lu J Elf S. Inhibition of human copper trafficking by a small molecule significantly attenuates cancer cell proliferation. Nat Chem (2015) 7:968–79. doi: 10.1038/nchem.2381 PMC4725056 26587712

[88] Tsang T Posimo JM Gudiel AA Cicchini M Feldser DM Brady DC. Copper is an essential regulator of the autophagic kinases ULK1/2 to drive lung adenocarcinoma. Nat Cell Biol (2020) 22:412–24. doi: 10.1038/s41556-020-0481-4 PMC7610258 32203415

[89] Polishchuk EV Merolla A Lichtmannegger J Romano A Indrieri A Ilyechova EY. Activation of autophagy, observed in liver tissues from patients with Wilson disease and from ATP7B-deficient animals, protects hepatocytes from copper-induced apoptosis. Gastroenterology (2019) 156:1173–1189.e5. doi: 10.1053/j.gastro.2018.11.032 30452922

[90] Aubert L Nandagopal N Steinhart Z Lavoie G Nourreddine S Berman J. Copper bioavailability is a KRAS-specific vulnerability in colorectal cancer. Nat Commun (2020) 11:3701. doi: 10.1038/s41467-020-17549-y 32709883 PMC7381612

[91] McAuslan BR Reilly W. Endothelial cell phagokinesis in response to specific metal ions. Exp Cell Res (1980) 130:147–57. doi: 10.1016/0014-4827(80)90051-8 6161014

[92] Ziche M Jones J Gullino PM. Role of prostaglandin E1 and copper in angiogenesis. J Natl Cancer Inst (1982) 69:475–82. 6180207

[93] Raju KS Alessandri G Ziche M Gullino PM. Ceruloplasmin, copper ions, and angiogenesis. J Natl Cancer Inst (1982) 69:1183–8. 6182332

[94] Soncin F Guitton JD Cartwright T Badet J. Interaction of human angiogenin with copper modulates angiogenin binding to endothelial cells. Biochem Biophys Res Commun (1997) 236:604–10. doi: 10.1006/bbrc.1997.7018 9245697

[95] Urso E Maffia M. Behind the link between copper and angiogenesis: established mechanisms and an overview on the role of vascular copper transport systems. J Vasc Res (2015) 52:172–96. doi: 10.1159/000438485 26484858

[96] Mandinov L Mandinova A Kyurkchiev S Kyurkchiev D Kehayov I Kolev V. Copper chelation represses the vascular response to injury. Proc Natl Acad Sci U.S.A. (2003) 100:6700–5. doi: 10.1073/pnas.1231994100 PMC164510 12754378

[97] Prudovsky I Bagala C Tarantini F Mandinova A Soldi R Bellum S. The intracellular translocation of the components of the fibroblast growth factor 1 release complex precedes their assembly prior to export. J Cell Biol (2002) 158:201–8. doi: 10.1083/jcb.200203084 PMC2173119 12135982

[98] Pan Q Kleer CG van Golen KL Irani J Bottema KM Bias C. Copper deficiency induced by tetrathiomolybdate suppresses tumor growth and angiogenesis. Cancer Res (2002) 62:4854–9. 12208730

[99] Kohno T Urao N Ashino T Sudhahar V McKinney RD Hamakubo T. Novel role of copper transport protein antioxidant-1 in neointimal formation after vascular injury. Arterioscler Thromb Vasc Biol (2013) 33:805–13. doi: 10.1161/ATVBAHA.112.300862 PMC3600157 23349186

[100] Zimna A Kurpisz M. Hypoxia-inducible factor-1 in physiological and pathophysiological angiogenesis: applications and therapies. BioMed Res Int (2015) 2015:549412. doi: 10.1155/2015/549412 26146622 PMC4471260

[101] Feng W Ye F Xue W Zhou Z Kang YJ. Copper regulation of hypoxia-inducible factor-1 activity. Mol Pharmacol (2009) 75:174–82. doi: 10.1124/mol.108.051516 PMC2685058 18842833

[102] Wu Z Zhang W Kang YJ. Copper affects the binding of HIF-1α to the critical motifs of its target genes. Metallomics (2019) 11:429–38. doi: 10.1039/c8mt00280k 30566157

[103] Himoto T Fujita K Nomura T Tani J Miyoshi H Morishita A. Roles of copper in hepatocarcinogenesis via the activation of hypoxia-inducible factor-1α. Biol Trace Elem Res (2016) 174:58–64. doi: 10.1007/s12011-016-0702-7 27121973

[104] Karginova O Weekley CM Raoul A Alsayed A Wu T Lee SS-Y. Inhibition of copper transport induces apoptosis in triple-negative breast cancer cells and suppresses tumor angiogenesis. Mol Cancer Ther (2019) 18:873–85. doi: 10.1158/1535-7163.MCT-18-0667 PMC12244435 30824611

[105] Bhuvanasundar R John A Sulochana KN Coral K Deepa PR Umashankar V. A molecular model of human lysyl oxidase (LOX) with optimal copper orientation in the catalytic cavity for induced fit docking studies with potential modulators. Bioinformation (2014) 10:406–12. doi: 10.6026/97320630010406 PMC4135287 25187679

[106] Erler JT Bennewith KL Cox TR Lang G Bird D Koong A. Hypoxia-induced lysyl oxidase is a critical mediator of bone marrow cell recruitment to form the premetastatic niche. Cancer Cell (2009) 15:35–44. doi: 10.1016/j.ccr.2008.11.012 19111879 PMC3050620

[107] Gacheru SN Trackman PC Shah MA O’Gara CY Spacciapoli P Greenaway FT. Structural and catalytic properties of copper in lysyl oxidase. J Biol Chem (1990) 265:19022–7. doi: 10.1016/S0021-9258(17)30618-X 1977746

[108] Shanbhag V Jasmer-McDonald K Zhu S Martin AL Gudekar N Khan A. ATP7A delivers copper to the lysyl oxidase family of enzymes and promotes tumorigenesis and metastasis. Proc Natl Acad Sci U.S.A. (2019) 116:6836–41. doi: 10.1073/pnas.1817473116 PMC6452744 30890638

[109] Barker HE Erler JT. The potential for LOXL2 as a target for future cancer treatment. Future Oncol (2011) 7:707–10. doi: 10.2217/fon.11.46 21675833

[110] Barker HE Chang J Cox TR Lang G Bird D Nicolau M. LOXL2-mediated matrix remodeling in metastasis and mammary gland involution. Cancer Res (2011) 71:1561–72. doi: 10.1158/0008-5472.CAN-10-2868 PMC3842018 21233336

[111] Peinado H Del Carmen Iglesias-de la Cruz M Olmeda D Csiszar K Fong KSK Vega S. A molecular role for lysyl oxidase-like 2 enzyme in snail regulation and tumor progression. EMBO J (2005) 24:3446–58. doi: 10.1038/sj.emboj.7600781 PMC1276164 16096638

[112] MacDonald G Nalvarte I Smirnova T Vecchi M Aceto N Dolemeyer A. Memo is a copper-dependent redox protein with an essential role in migration and metastasis. Sci Signal (2014) 7:ra56. doi: 10.1126/scisignal.2004870 24917593

[113] Li S Zhang J Yang H Wu C Dang X Liu Y. Copper depletion inhibits CoCl2-induced aggressive phenotype of MCF-7 cells via downregulation of HIF-1 and inhibition of Snail/Twist-mediated epithelial-mesenchymal transition. Sci Rep (2015) 5:12410. doi: 10.1038/srep12410 26174737 PMC4502431

[114] Schulze A Harris AL. How cancer metabolism is tuned for proliferation and vulnerable to disruption. Nature (2012) 491:364–73. doi: 10.1038/nature11706 23151579

[115] Semenza GL. Hypoxia-inducible factor 1 (HIF-1) pathway. Sci STKE (2007) 2007:cm8. doi: 10.1126/stke.4072007cm8 17925579

[116] Martin F Linden T Katschinski DM Oehme F Flamme I Mukhopadhyay CK. Copper-dependent activation of hypoxia-inducible factor (HIF)-1: implications for ceruloplasmin regulation. Blood (2005) 105:4613–9. doi: 10.1182/blood-2004-10-3980 15741220

[117] Lv B Wang Y Ma D Cheng W Liu J Yong T. Immunotherapy: reshape the tumor immune microenvironment. Front Immunol (2022) 13:844142. doi: 10.3389/fimmu.2022.844142 35874717 PMC9299092

[118] Crowe A Jackaman C Beddoes KM Ricciardo B Nelson DJ. Rapid copper acquisition by developing murine mesothelioma: decreasing bioavailable copper slows tumor growth, normalizes vessels and promotes T cell infiltration. PloS One (2013) 8:e73684. doi: 10.1371/journal.pone.0073684 24013775 PMC3754934

[119] Voli F Valli E Lerra L Kimpton K Saletta F Giorgi FM. Intratumoral copper modulates PD-L1 expression and influences tumor immune evasion. Cancer Res (2020) 80:4129–44. doi: 10.1158/0008-5472.CAN-20-0471 32816860

[120] Chakraborty P Das S Banerjee K Sinha A Roy S Chatterjee M. A copper chelate selectively triggers apoptosis in myeloid-derived suppressor cells in a drug-resistant tumor model and enhances antitumor immune response. Immunopharmacol Immunotoxicol (2014) 36:165–75. doi: 10.3109/08923973.2014.897727 24611750

[121] Serra M Columbano A Ammarah U Mazzone M Menga A. Understanding metal dynamics between cancer cells and macrophages: competition or synergism? Front Oncol (2020) 10:646. doi: 10.3389/fonc.2020.00646 32426284 PMC7203474

[122] Stern BR. Essentiality and toxicity in copper health risk assessment: overview, update and regulatory considerations. J Toxicol Environ Health A (2010) 73:114–27. doi: 10.1080/15287390903337100 20077283

[123] Scheiber I Dringen R Mercer JFB. Copper: effects of deficiency and overload. Met Ions Life Sci (2013) 13:359–87. doi: 10.1007/978-94-007-7500-8_11 24470097

[124] Aggarwal A Bhatt M. Advances in treatment of Wilson disease. Tremor Other Hyperkinet Mov (N Y) (2018) 8:525. doi: 10.7916/D841881D 29520330 PMC5840318

[125] Gromadzka G Tarnacka B Flaga A Adamczyk A. Copper dyshomeostasis in neurodegenerative diseases-therapeutic implications. Int J Mol Sci (2020) 21:9259. doi: 10.3390/ijms21239259 33291628 PMC7730516

[126] Davies KM Mercer JFB Chen N Double KL. Copper dyshomoeostasis in parkinson’s disease: implications for pathogenesis and indications for novel therapeutics. Clin Sci (Lond) (2016) 130:565–74. doi: 10.1042/CS20150153 26957644

[127] Nirmala JG Lopus M. Cell death mechanisms in eukaryotes. Cell Biol Toxicol (2020) 36:145–64. doi: 10.1007/s10565-019-09496-2 31820165

[128] Yang F Pei R Zhang Z Liao J Yu W Qiao N. Copper induces oxidative stress and apoptosis through mitochondria-mediated pathway in chicken hepatocytes. Toxicol In Vitro (2019) 54:310–6. doi: 10.1016/j.tiv.2018.10.017 30389602

[129] Nagai M Vo NH Shin Ogawa L Chimmanamada D Inoue T Chu J. The oncology drug elesclomol selectively transports copper to the mitochondria to induce oxidative stress in cancer cells. Free Radic Biol Med (2012) 52:2142–50. doi: 10.1016/j.freeradbiomed.2012.03.017 22542443

[130] Israels LG Israels ED. Apoptosis. Stem Cells (1999) 17:306–13. doi: 10.1002/stem.170306 10527465

[131] Cen D Brayton D Shahandeh B Meyskens FL Farmer PJ. Disulfiram facilitates intracellular Cu uptake and induces apoptosis in human melanoma cells. J Med Chem (2004) 47:6914–20. doi: 10.1021/jm049568z 15615540

[132] Kirshner JR He S Balasubramanyam V Kepros J Yang C-Y Zhang M. Elesclomol induces cancer cell apoptosis through oxidative stress. Mol Cancer Ther (2008) 7:2319–27. doi: 10.1158/1535-7163.MCT-08-0298 18723479

[133] Wu H Guo H Liu H Cui H Fang J Zuo Z. Copper sulfate-induced endoplasmic reticulum stress promotes hepatic apoptosis by activating CHOP, JNK and caspase-12 signaling pathways. Ecotoxicol Environ Saf (2020) 191:110236. doi: 10.1016/j.ecoenv.2020.110236 32001424

[134] Liu H Guo H Jian Z Cui H Fang J Zuo Z. Copper induces oxidative stress and apoptosis in the mouse liver. Oxid Med Cell Longev (2020) 2020:1359164. doi: 10.1155/2020/1359164 32411316 PMC7201649

[135] Luo Q Song Y Kang J Wu Y Wu F Li Y. mtROS-mediated Akt/AMPK/mTOR pathway was involved in copper-induced autophagy and it attenuates copper-induced apoptosis in RAW264.7 mouse monocytes. Redox Biol (2021) 41:101912. doi: 10.1016/j.redox.2021.101912 33706171 PMC7944049

[136] Yang F Liao J Yu W Qiao N Guo J Han Q. Exposure to copper induces mitochondria-mediated apoptosis by inhibiting mitophagy and the PINK1/parkin pathway in chicken (Gallus gallus) livers. J Hazard Mater (2021) 408:124888. doi: 10.1016/j.jhazmat.2020.124888 33360697

[137] Zhao G Sun H Zhang T Liu J-X. Copper induce zebrafish retinal developmental defects via triggering stresses and apoptosis. Cell Commun Signal (2020) 18:45. doi: 10.1186/s12964-020-00548-3 32169084 PMC7071659

[138] Tardito S Barilli A Bassanetti I Tegoni M Bussolati O Franchi-Gazzola R. Copper-dependent cytotoxicity of 8-hydroxyquinoline derivatives correlates with their hydrophobicity and does not require caspase activation. J Med Chem (2012) 55:10448–59. doi: 10.1021/jm301053a 23170953

[139] Buccarelli M D’Alessandris QG Matarrese P Mollinari C Signore M Cappannini A. Elesclomol-induced increase of mitochondrial reactive oxygen species impairs glioblastoma stem-like cell survival and tumor growth. J Exp Clin Cancer Res (2021) 40:228. doi: 10.1186/s13046-021-02031-4 34253243 PMC8273992

[140] Gong Y Fan Z Luo G Yang C Huang Q Fan K. The role of necroptosis in cancer biology and therapy. Mol Cancer (2019) 18:100. doi: 10.1186/s12943-019-1029-8 31122251 PMC6532150

[141] Xue C Gu X Li G Bao Z Li L. Mitochondrial mechanisms of necroptosis in liver diseases. Int J Mol Sci (2020) 22:66. doi: 10.3390/ijms22010066 33374660 PMC7793526

[142] Sepand M-R Aliomrani M Hasani-Nourian Y Khalhori M-R Farzaei M-H Sanadgol N. Mechanisms and pathogenesis underlying environmental chemical-induced necroptosis. Environ Sci pollut Res Int (2020) 27:37488–501. doi: 10.1007/s11356-020-09360-5 32683625

[143] Krumschnabel G Ebner HL Hess MW Villunger A. Apoptosis and necroptosis are induced in rainbow trout cell lines exposed to cadmium. Aquat Toxicol (2010) 99:73–85. doi: 10.1016/j.aquatox.2010.04.005 20435356

[144] Chen W Wang X Zhao B Zhang R Xie Z He Y. CuS-MnS2 nano-flowers for magnetic resonance imaging guided photothermal/photodynamic therapy of ovarian cancer through necroptosis. Nanoscale (2019) 11:12983–9. doi: 10.1039/c9nr03114f 31264665

[145] Lan Y Bai P Liu Y Afshar S Striar R Rattray AK. Visualization of receptor-interacting protein kinase 1 (RIPK1) by brain imaging with positron emission tomography. J Med Chem (2021) 64:15420–8. doi: 10.1021/acs.jmedchem.1c01477 PMC8858444 34652135

[146] Kovacs SB Miao EA. Gasdermins: effectors of pyroptosis. Trends Cell Biol (2017) 27:673–84. doi: 10.1016/j.tcb.2017.05.005 PMC5565696 28619472

[147] Deigendesch N Zychlinsky A Meissner F. Copper regulates the canonical NLRP3 inflammasome. J Immunol (2018) 200:1607–17. doi: 10.4049/jimmunol.1700712 29358279

[148] Tao X Wan X Wu D Song E Song Y. A tandem activation of NLRP3 inflammasome induced by copper oxide nanoparticles and dissolved copper ion in J774A.1 macrophage. J Hazard Mater (2021) 411:125134. doi: 10.1016/j.jhazmat.2021.125134 33485222

[149] Liao J Yang F Tang Z Yu W Han Q Hu L. Inhibition of caspase-1-dependent pyroptosis attenuates copper-induced apoptosis in chicken hepatocytes. Ecotoxicol Environ Saf (2019) 174:110–9. doi: 10.1016/j.ecoenv.2019.02.069 30822667

[150] Dong J Wang X Xu C Gao M Wang S Zhang J. Inhibiting NLRP3 inflammasome activation prevents copper-induced neuropathology in a murine model of wilson’s disease. Cell Death Dis (2021) 12:87. doi: 10.1038/s41419-021-03397-1 33462188 PMC7813851

[151] Liao J Hu Z Li Q Li H Chen W Huo H. Endoplasmic reticulum stress contributes to copper-induced pyroptosis via regulating the IRE1α-XBP1 pathway in pig jejunal epithelial cells. J Agric Food Chem (2022) 70:1293–303. doi: 10.1021/acs.jafc.1c07927 35075900

[152] Hufnagel M Neuberger R Wall J Link M Friesen A Hartwig A. Impact of differentiated macrophage-like cells on the transcriptional toxicity profile of CuO nanoparticles in Co-cultured lung epithelial cells. Int J Mol Sci (2021) 22:5044. doi: 10.3390/ijms22095044 34068728 PMC8126233

[153] Gan B. Mitochondrial regulation of ferroptosis. J Cell Biol (2021) 220:e202105043. doi: 10.1083/jcb.202105043 34328510 PMC8329737

[154] Gao W Huang Z Duan J Nice EC Lin J Huang C. Elesclomol induces copper-dependent ferroptosis in colorectal cancer cells via degradation of ATP7A. Mol Oncol (2021) 15:3527–44. doi: 10.1002/1878-0261.13079 PMC8637554 34390123

[155] Rakshit A Khatua K Shanbhag V Comba P Datta A. Cu2+ selective chelators relieve copper-induced oxidative stress *in vivo* . Chem Sci (2018) 9:7916–30. doi: 10.1039/c8sc04041a PMC6202919 30450181

[156] Maher P. Potentiation of glutathione loss and nerve cell death by the transition metals iron and copper: implications for age-related neurodegenerative diseases. Free Radic Biol Med (2018) 115:92–104. doi: 10.1016/j.freeradbiomed.2017.11.015 29170091

[157] Guo H Ouyang Y Yin H Cui H Deng H Liu H. Induction of autophagy via the ROS-dependent AMPK-mTOR pathway protects copper-induced spermatogenesis disorder. Redox Biol (2022) 49:102227. doi: 10.1016/j.redox.2021.102227 34979450 PMC8728583

[158] Lei G Zhuang L Gan B. Targeting ferroptosis as a vulnerability in cancer. Nat Rev Cancer (2022) 22:381–96. doi: 10.1038/s41568-022-00459-0 PMC10243716 35338310

[159] Li Y Chen F Chen J Chan S He Y Liu W. Disulfiram/Copper induces antitumor activity against both nasopharyngeal cancer cells and cancer-associated fibroblasts through ROS/MAPK and ferroptosis pathways. Cancers (Basel) (2020) 12:138. doi: 10.3390/cancers12010138 31935835 PMC7017005

[160] Ren X Li Y Zhou Y Hu W Yang C Jing Q. Overcoming the compensatory elevation of NRF2 renders hepatocellular carcinoma cells more vulnerable to disulfiram/copper-induced ferroptosis. Redox Biol (2021) 46:102122. doi: 10.1016/j.redox.2021.102122 34482117 PMC8416961

[161] Tang X Ren X Huang T Miao Y Ha W Li Z. Prognostic and immunological significance of the molecular subtypes and risk signatures based on cuproptosis in hepatocellular carcinoma. Mediators Inflammation (2023) 2023:3951940. doi: 10.1155/2023/3951940 PMC10139815 37124062

[162] Yang W Wang Y Huang Y Yu J Wang T Li C. 4-octyl itaconate inhibits aerobic glycolysis by targeting GAPDH to promote cuproptosis in colorectal cancer. BioMed Pharmacother (2023) 159:114301. doi: 10.1016/j.biopha.2023.114301 36706634

[163] Guo B Yang F Zhang L Zhao Q Wang W Yin L. Cuproptosis induced by ROS responsive nanoparticles with elesclomol and copper combined with αPD-L1 for enhanced cancer immunotherapy. Adv Mater (2023) 35:e2212267. doi: 10.1002/adma.202212267 36916030

[164] Hanahan D Weinberg RA. Hallmarks of cancer: the next generation. Cell (2011) 144:646–74. doi: 10.1016/j.cell.2011.02.013 21376230

[165] Daniel KG Gupta P Harbach RH Guida WC Dou QP. Organic copper complexes as a new class of proteasome inhibitors and apoptosis inducers in human cancer cells. Biochem Pharmacol (2004) 67:1139–51. doi: 10.1016/j.bcp.2003.10.031 15006550

[166] Tawari PE Wang Z Najlah M Tsang CW Kannappan V Liu P. The cytotoxic mechanisms of disulfiram and copper(ii) in cancer cells. Toxicol Res (Camb) (2015) 4:1439–42. doi: 10.1039/c5tx00210a PMC5027600 27708770

[167] Brewer GJ. The promise of copper lowering therapy with tetrathiomolybdate in the cure of cancer and in the treatment of inflammatory disease. J Trace Elem Med Biol (2014) 28:372–8. doi: 10.1016/j.jtemb.2014.07.015 25194954

[168] Song MO Li J Freedman JH. Physiological and toxicological transcriptome changes in HepG2 cells exposed to copper. Physiol Genomics (2009) 38:386–401. doi: 10.1152/physiolgenomics.00083.2009 19549813 PMC3774564

[169] Harro CC Smedley RC Buchweitz JP Langlois DK. Hepatic copper and other trace mineral concentrations in dogs with hepatocellular carcinoma. J Vet Intern Med (2019) 33:2193–9. doi: 10.1111/jvim.15619 PMC6766484 31493348

[170] Tamai Y Iwasa M Eguchi A Shigefuku R Sugimoto K Hasegawa H. Serum copper, zinc and metallothionein serve as potential biomarkers for hepatocellular carcinoma. PloS One (2020) 15:e0237370. doi: 10.1371/journal.pone.0237370 32857769 PMC7455040

[171] Kim J-J Kim Y-S Kumar V. Heavy metal toxicity: an update of chelating therapeutic strategies. J Trace Elements Med Biol (2019) 54:226–31. doi: 10.1016/j.jtemb.2019.05.003 31109617

[172] Davis CI Gu X Kiefer RM Ralle M Gade TP Brady DC. Altered copper homeostasis underlies sensitivity of hepatocellular carcinoma to copper chelation. Metallomics (2020) 12:1995–2008. doi: 10.1039/d0mt00156b 33146201 PMC8315290

[173] Pan Q Rosenthal DT Bao L Kleer CG Merajver SD. Antiangiogenic tetrathiomolybdate protects against Her2/neu-induced breast carcinoma by hypoplastic remodeling of the mammary gland. Clin Cancer Res (2009) 15:7441–6. doi: 10.1158/1078-0432.CCR-09-1361 PMC3471244 19934283

[174] Brady DC Crowe MS Turski ML Hobbs GA Yao X Chaikuad A. Copper is required for oncogenic BRAF signalling and tumorigenesis. Nature (2014) 509:492–6. doi: 10.1038/nature13180 PMC4138975 24717435

[175] Chan N Willis A Kornhauser N Ward MM Lee SB Nackos E. Influencing the tumor microenvironment: a phase II study of copper depletion using tetrathiomolybdate in patients with breast cancer at high risk for recurrence and in preclinical models of lung metastases. Clin Cancer Res (2017) 23:666–76. doi: 10.1158/1078-0432.CCR-16-1326 27769988

[176] Bryant KL Stalnecker CA Zeitouni D Klomp JE Peng S Tikunov AP. Combination of ERK and autophagy inhibition as a treatment approach for pancreatic cancer. Nat Med (2019) 25:628–40. doi: 10.1038/s41591-019-0368-8 PMC6484853 30833752

[177] Krishnan N Felice C Rivera K Pappin DJ Tonks NK. DPM-1001 decreased copper levels and ameliorated deficits in a mouse model of wilson’s disease. Genes Dev (2018) 32:944–52. doi: 10.1101/gad.314658.118 PMC6075031 29945887

[178] Ishida S McCormick F Smith-McCune K Hanahan D. Enhancing tumor-specific uptake of the anticancer drug cisplatin with a copper chelator. Cancer Cell (2010) 17:574–83. doi: 10.1016/j.ccr.2010.04.011 PMC2902369 20541702

[179] Cui L Gouw AM LaGory EL Guo S Attarwala N Tang Y. Mitochondrial copper depletion suppresses triple-negative breast cancer in mice. Nat Biotechnol (2021) 39:357–67. doi: 10.1038/s41587-020-0707-9 PMC7956242 33077961

[180] Ramchandani D Berisa M Tavarez DA Li Z Miele M Bai Y. Copper depletion modulates mitochondrial oxidative phosphorylation to impair triple negative breast cancer metastasis. Nat Commun (2021) 12:7311. doi: 10.1038/s41467-021-27559-z 34911956 PMC8674260

[181] Jain S Cohen J Ward MM Kornhauser N Chuang E Cigler T. Tetrathiomolybdate-associated copper depletion decreases circulating endothelial progenitor cells in women with breast cancer at high risk of relapse. Ann Oncol (2013) 24:1491–8. doi: 10.1093/annonc/mds654 PMC3707432 23406736

[182] Tessmer CF Hrgovcic M Thomas FB Fuller LM Castro JR. Serum copper as an index of tumor response to radiotherapy. Radiology (1973) 106:635–9. doi: 10.1148/106.3.635 4734340

[183] Yang M Wu X Hu J Wang Y Wang Y Zhang L. COMMD10 inhibits HIF1α/CP loop to enhance ferroptosis and radiosensitivity by disrupting Cu-fe balance in hepatocellular carcinoma. J Hepatol (2022) 76:1138–50. doi: 10.1016/j.jhep.2022.01.009 35101526

[184] Walshe JM. Treatment of wilson’s disease with trientine (triethylene tetramine) dihydrochloride. Lancet (1982) 1:643–7. doi: 10.1016/s0140-6736(82)92201-2 6121964

[185] Trachootham D Alexandre J Huang P. Targeting cancer cells by ROS-mediated mechanisms: a radical therapeutic approach? Nat Rev Drug Discovery (2009) 8:579–91. doi: 10.1038/nrd2803 19478820

[186] Fruehauf JP Meyskens FL. Reactive oxygen species: a breath of life or death? Clin Cancer Res (2007) 13:789–94. doi: 10.1158/1078-0432.CCR-06-2082 17289868

[187] Shimada K Reznik E Stokes ME Krishnamoorthy L Bos PH Song Y. Copper-binding small molecule induces oxidative stress and cell cycle arrest in glioblastoma-patient-derived cells. Cell Chem Biol (2018) 25:585–594.e7. doi: 10.1016/j.chembiol.2018.02.010 29576531 PMC5959763

[188] Kannappan V Ali M Small B Rajendran G Elzhenni S Taj H. Recent advances in repurposing disulfiram and disulfiram derivatives as copper-dependent anticancer agents. Front Mol Biosci (2021) 8:741316. doi: 10.3389/fmolb.2021.741316 34604310 PMC8484884

[189] Ekinci E Rohondia S Khan R Dou QP. Repurposing disulfiram as an anti-cancer agent: updated review on literature and patents. Recent Pat Anticancer Drug Discovery (2019) 14:113–32. doi: 10.2174/1574892814666190514104035 31084595

[190] Kona FR Buac D M Burger A. Disulfiram, and disulfiram derivatives as novel potential anticancer drugs targeting the ubiquitin-proteasome system in both preclinical and clinical studies. Curr Cancer Drug Targets (2011) 11:338–46. doi: 10.2174/156800911794519798 21247383

[191] Li Y Wang L-H Zhang H-T Wang Y-T Liu S Zhou W-L. Disulfiram combined with copper inhibits metastasis and epithelial-mesenchymal transition in hepatocellular carcinoma through the NF-κB and TGF-β pathways. J Cell Mol Med (2018) 22:439–51. doi: 10.1111/jcmm.13334 PMC5742719 29148232

[192] Xu B Wang S Li R Chen K He L Deng M. Disulfiram/copper selectively eradicates AML leukemia stem cells *in vitro* and *in vivo* by simultaneous induction of ROS-JNK and inhibition of NF-κB and Nrf2. Cell Death Dis (2017) 8:e2797. doi: 10.1038/cddis.2017.176 28518151 PMC5520701

[193] Sun T Yang W Toprani SM Guo W He L DeLeo AB. Induction of immunogenic cell death in radiation-resistant breast cancer stem cells by repurposing anti-alcoholism drug disulfiram. Cell Commun Signal (2020) 18:36. doi: 10.1186/s12964-019-0507-3 32138738 PMC7057578

[194] Serra R Zhao T Huq S Gorelick NL Casaos J Cecia A. Disulfiram and copper combination therapy targets NPL4, cancer stem cells and extends survival in a medulloblastoma model. PloS One (2021) 16:e0251957. doi: 10.1371/journal.pone.0251957 34731160 PMC8565761

[195] Yip NC Fombon IS Liu P Brown S Kannappan V Armesilla AL. Disulfiram modulated ROS-MAPK and NFκB pathways and targeted breast cancer cells with cancer stem cell-like properties. Br J Cancer (2011) 104:1564–74. doi: 10.1038/bjc.2011.126 PMC3101904 21487404

[196] Chen D Cui QC Yang H Dou QP. Disulfiram, a clinically used anti-alcoholism drug and copper-binding agent, induces apoptotic cell death in breast cancer cultures and xenografts via inhibition of the proteasome activity. Cancer Res (2006) 66:10425–33. doi: 10.1158/0008-5472.CAN-06-2126 17079463

[197] Liu N Huang H Dou QP Liu J. Inhibition of 19S proteasome-associated deubiquitinases by metal-containing compounds. Oncoscience (2015) 2:457–66. doi: 10.18632/oncoscience.167 PMC4468331 26097878

[198] Skrott Z Mistrik M Andersen KK Friis S Majera D Gursky J. Alcohol-abuse drug disulfiram targets cancer via p97 segregase adaptor NPL4. Nature (2017) 552:194–9. doi: 10.1038/nature25016 PMC5730499 29211715

[199] Zhang G Wang Y Fuchs BC Guo W Drum DL Erstad DJ. Improving the therapeutic efficacy of sorafenib for hepatocellular carcinoma by repurposing disulfiram. Front Oncol (2022) 12:913736. doi: 10.3389/fonc.2022.913736 35912209 PMC9329590

[200] Gao X Huang H Pan C Mei Z Yin S Zhou L. Disulfiram/Copper induces immunogenic cell death and enhances CD47 blockade in hepatocellular carcinoma. Cancers (Basel) (2022) 14:4715. doi: 10.3390/cancers14194715 36230638 PMC9564202

[201] McMahon A Chen W Li F. Old wine in new bottles: advanced drug delivery systems for disulfiram-based cancer therapy. J Control Release (2020) 319:352–9. doi: 10.1016/j.jconrel.2020.01.001 31911155

[202] Fukai T Ushio-Fukai M Kaplan JH. Copper transporters and copper chaperones: roles in cardiovascular physiology and disease. Am J Physiol Cell Physiol (2018) 315:C186–201. doi: 10.1152/ajpcell.00132.2018 PMC6139499 29874110

[203] Trachootham D Zhou Y Zhang H Demizu Y Chen Z Pelicano H. Selective killing of oncogenically transformed cells through a ROS-mediated mechanism by beta-phenylethyl isothiocyanate. Cancer Cell (2006) 10:241–52. doi: 10.1016/j.ccr.2006.08.009 16959615

[204] Schumacker PT. Reactive oxygen species in cancer cells: live by the sword, die by the sword. Cancer Cell (2006) 10:175–6. doi: 10.1016/j.ccr.2006.08.015 16959608

[205] Lee JH Cho YS Jung K-H Park JW Lee K-H. Genipin enhances the antitumor effect of elesclomol in A549 lung cancer cells by blocking uncoupling protein-2 and stimulating reactive oxygen species production. Oncol Lett (2020) 20:374. doi: 10.3892/ol.2020.12237 33154772 PMC7608048

[206] Rushworth GF Megson IL. Existing and potential therapeutic uses for n-acetylcysteine: the need for conversion to intracellular glutathione for antioxidant benefits. Pharmacol Ther (2014) 141:150–9. doi: 10.1016/j.pharmthera.2013.09.006 24080471

[207] Wangpaichitr M Wu C You M Maher JC Dinh V Feun LG. N’,N’-Dimethyl-N’,N’-bis(phenylcarbonothioyl) propanedihydrazide (Elesclomol) selectively kills cisplatin resistant lung cancer cells through reactive oxygen species (ROS). Cancers (Basel) (2009) 1:23–38. doi: 10.3390/cancers1010023 20535236 PMC2882109

[208] Modica-Napolitano JS Bharath LP Hanlon AJ Hurley LD. The anticancer agent elesclomol has direct effects on mitochondrial bioenergetic function in isolated mammalian mitochondria. Biomolecules (2019) 9:298. doi: 10.3390/biom9080298 31344923 PMC6724019

[209] Hasinoff BB Wu X Yadav AA Patel D Zhang H Wang D-S. Cellular mechanisms of the cytotoxicity of the anticancer drug elesclomol and its complex with Cu(II). Biochem Pharmacol (2015) 93:266–76. doi: 10.1016/j.bcp.2014.12.008 25550273

[210] Tsvetkov P Detappe A Cai K Keys HR Brune Z Ying W. Mitochondrial metabolism promotes adaptation to proteotoxic stress. Nat Chem Biol (2019) 15:681–9. doi: 10.1038/s41589-019-0291-9 PMC8183600 31133756

[211] Kluza J Corazao-Rozas P Touil Y Jendoubi M Maire C Guerreschi P. Inactivation of the HIF-1α/PDK3 signaling axis drives melanoma toward mitochondrial oxidative metabolism and potentiates the therapeutic activity of pro-oxidants. Cancer Res (2012) 72:5035–47. doi: 10.1158/0008-5472.CAN-12-0979 22865452

[212] O’Day S Gonzalez R Lawson D Weber R Hutchins L Anderson C. Phase II, randomized, controlled, double-blinded trial of weekly elesclomol plus paclitaxel versus paclitaxel alone for stage IV metastatic melanoma. J Clin Oncol (2009) 27:5452–8. doi: 10.1200/JCO.2008.17.1579 19826135

[213] O’Day SJ Eggermont AMM Chiarion-Sileni V Kefford R Grob JJ Mortier L. Final results of phase III SYMMETRY study: randomized, double-blind trial of elesclomol plus paclitaxel versus paclitaxel alone as treatment for chemotherapy-naive patients with advanced melanoma. J Clin Oncol (2013) 31:1211–8. doi: 10.1200/JCO.2012.44.5585 23401447

[214] Berkenblit A Eder JP Ryan DP Seiden MV Tatsuta N Sherman ML. Phase I clinical trial of STA-4783 in combination with paclitaxel in patients with refractory solid tumors. Clin Cancer Res (2007) 13:584–90. doi: 10.1158/1078-0432.CCR-06-0964 17255281

[215] Hedley D Shamas-Din A Chow S Sanfelice D Schuh AC Brandwein JM. A phase I study of elesclomol sodium in patients with acute myeloid leukemia. Leuk Lymphoma (2016) 57:2437–40. doi: 10.3109/10428194.2016.1138293 26732437

[216] Zheng P Zhou C Lu L Liu B Ding Y. Elesclomol: a copper ionophore targeting mitochondrial metabolism for cancer therapy. J Exp Clin Cancer Res (2022) 41:271. doi: 10.1186/s13046-022-02485-0 36089608 PMC9465867

